# Ping-Pong—Tumor and Host in Pancreatic Cancer Progression

**DOI:** 10.3389/fonc.2019.01359

**Published:** 2019-12-16

**Authors:** Wei Mu, Zhe Wang, Margot Zöller

**Affiliations:** ^1^School of Public Health, Shanghai Jiao Tong University School of Medicine, Shanghai, China; ^2^Department of Oncology, The First Affiliated Hospital of Guangdong, Pharmaceutical University, Guangzhou, China

**Keywords:** pancreatic cancer, metastasis, exosomes, cancer-initiating cell markers, stellate cells, metabolism, perineural invasion, immunosuppression

## Abstract

Metastasis is the main cause of high pancreatic cancer (PaCa) mortality and trials dampening PaCa mortality rates are not satisfying. Tumor progression is driven by the crosstalk between tumor cells, predominantly cancer-initiating cells (CIC), and surrounding cells and tissues as well as distant organs, where tumor-derived extracellular vesicles (TEX) are of major importance. A strong stroma reaction, recruitment of immunosuppressive leukocytes, perineural invasion, and early spread toward the peritoneal cavity, liver, and lung are shared with several epithelial cell-derived cancer, but are most prominent in PaCa. Here, we report on the state of knowledge on the PaCIC markers Tspan8, alpha6beta4, CD44v6, CXCR4, LRP5/6, LRG5, claudin7, EpCAM, and CD133, which all, but at different steps, are engaged in the metastatic cascade, frequently via PaCIC-TEX. This includes the contribution of PaCIC markers to TEX biogenesis, targeting, and uptake. We then discuss PaCa-selective features, where feedback loops between stromal elements and tumor cells, including distorted transcription, signal transduction, and metabolic shifts, establish vicious circles. For the latter particularly pancreatic stellate cells (PSC) are responsible, furnishing PaCa to cope with poor angiogenesis-promoted hypoxia by metabolic shifts and direct nutrient transfer via vesicles. Furthermore, nerves including Schwann cells deliver a large range of tumor cell attracting factors and Schwann cells additionally support PaCa cell survival by signaling receptor binding. PSC, tumor-associated macrophages, and components of the dysplastic stroma contribute to perineural invasion with signaling pathway activation including the cholinergic system. Last, PaCa aggressiveness is strongly assisted by the immune system. Although rich in immune cells, only immunosuppressive cells and factors are recovered in proximity to tumor cells and hamper effector immune cells entering the tumor stroma. Besides a paucity of immunostimulatory factors and receptors, immunosuppressive cytokines, myeloid-derived suppressor cells, regulatory T-cells, and M2 macrophages as well as PSC actively inhibit effector cell activation. This accounts for NK cells of the non-adaptive and cytotoxic T-cells of the adaptive immune system. We anticipate further deciphering the molecular background of these recently unraveled intermingled phenomena may turn most lethal PaCa into a curatively treatable disease.

## Introduction

### The Metastatic Cascade and Tumor Cell Dissemination

More than 90% of cancer mortality is related to metastasis (1), which in carcinoma requires completion of the metastatic cascade starting with local invasion of the surrounding extracellular matrix (ECM) and cells and processing through intravasation, surviving transport in vessels, arrest at distant organs, extravasation, surviving in the foreign environment and reinitiating tumor growth (2). These complex biological events are orchestrated by cell autonomous and non-autonomous signaling cascades. Local invasion requires breaching the basal membrane (BM) promoted by tumor-derived proteases and leading to liberation of growth factors and integrin activation affecting cell polarity and survival (3). Alternatively, tumor cells may use a protease- and integrin-independent, Rho^1^/ROCK^1^-dependent amoeboid invasion program (4). For local invasion of individual cells, tumor cells adopt a developmental epithelial-mesenchymal transition (EMT) program, which orchestrates activation of sets of transcription factors (Tf) that repress cell-cell adhesion molecules and induce expression of mesenchymal markers (5). Having passed the BM, tumor cells encounter the tumor stroma, which consists of endothelial cells (EC), pericytes, adipocytes, fibroblasts (FB), and bone marrow mesenchymal cells. Tumor cells push the reactive stroma toward pro-tumorigenic factor secretion and pro-tumorigenic cell recruitment. Thus, contact with the surrounding stroma is the first step where tumor cells receive a self-amplifying feedback (6, 7). The following step of invasion is strongly promoted by tumor-induced angiogenesis/lymphangiogenesis, the newly formed vessels being tortuous, leaky and continuously reconfiguring themselves, weak interactions between adjacent EC and the incomplete pericyte coverage facilitate tumor cell intravasation. EC wall passage is assisted by TGFβ^1^ and tumor-associated macrophages (TAM), providing CFS1/MCSF^1^ and EGF^1^. In addition, metabolic adaptations of growing and sprouting EC support (lymph)angiogenesis (8–10). In the vasculature, tumor cells are exposed to a variety of stresses. In the absence of cell-cell or cell-matrix adhesion, epithelial cell would undergo apoptosis/anoikis, which is circumvented by metabolic shifts toward the pentosephosphate pathway and anaerobic glycolysis. Matrix detachment-forced reduced glucose uptake assists LKB1^1^ activation,^1^ which increases protein kinase AMP^1^ catalytic subunit PRKAA^1^ activity. This inhibits acetyl-CoA carboxylases ACACA/B^1^, lowers NADPH^1^ consumption in fatty acid (FA) synthesis, but increases NADPH generation through an alternative pathway. This process reduces reactive oxygen species (ROS), essential for precluding detached cancer cell anoikis (10–13). Shear stress and the attack by the innate immune system are circumvented by tumor cell tissue factor (TF^1^) and selectins binding to platelets to form microemboli, which act as protective shields for the tumor cells (14, 15). Tumor cells mostly extravasate between adjacent EC. Adhesion to EC is facilitated by selectins, cadherins, integrins, CD44, Ig superfamily members, CD146/MUC18^1^, and by homophilic interactions between JAM^1^. Interactions between tumor cell-provided factors such as ANGPTL4^1^ and α5β1, CDH5/CD144^1^, CLDN5^1^, EREG^1^, COX2^1^, and MMP^1^ support extravasation. Actin remodeling, opening of junctions, necroptosis and APP^1^-DR6^1^-assisted EC death are discussed as underlying mechanisms. Platelet-, neutrophil- and monocyte-provided cytokines and chemokines also assist extravasation (16, 17).

### Metastatic Growth

There is ample evidence that migrating cancer cells leave the circulation for well-prepared soil, known as premetastatic niche. It is arranged in advance of cancer cell arrival by receiving information via tumor exosomes (TEX). Integrins, tetraspanins, receptor tyrosine kinases (RTK) and G-protein coupled receptors (GPCR) are important for message transfer (18–21). Established micrometastases may persist for weeks to years in a state of long-term dormancy. This dormancy relies on resting state persistence or failure to initiate angiogenesis, or on apoptosis-promoting host cells. Macroscopic metastatic outgrowth requires a multitude of adaptive programs that vary depending on the organ site of the metastasis and the original tumor. No metastasis-specific genetic changes being observed, outgrowth is supposed relying on epigenetic changes, like aberrant DNA methylation, altered chromatin structure, and activation of transcriptional programs that can be facilitated/guided by long non-coding (lnc)RNA. Two prerequisites must be fulfilled. One is the presence of cancer-initiating cells (CIC) with the capacity for self-renewal that in part is promoted by EMT-related Tf. The other is the establishment of adaptive programs enabling growth in the foreign environment. This includes some common traits such as metabolic adaptation and survival pathway activation. Other adaptive programs vary with the site of metastasis. Thus, similar to primary tumor growth, metastatic outgrowth is supported by the surrounding stroma including TGFβ1 and periostin, pro-inflammatory cells, local fibroblasts, and supportive ECM components (22–24). There remains a last query. CIC-derived metastases frequently reflect the mixed phenotype of the primary tumor. This may be due to the reversibility of EMT, called mesenchymal-epithelial transition (MET). However, further studies are required to elucidate tumor-inherent and surrounding-supported MET reprogramming (25–27).

Twenty-five years ago, the metastatic cascade was described as sequential processes in microecosystems (28). This still holds true, where striking progress in molecular characterization, important insights into stem cell (SC)/CIC plasticity, signaling pathways, networking connectivity and the modes of epigenetic regulation allowed deciphering the paths toward tumor progression.

After briefly introducing the clinical features of PaCa and exosome composition, we discuss current theories on the molecular mechanisms underlying the steps of the metastatic cascade particularly in PaCa.

## Clinical Features of Pancreatic Cancer Growth and Metastasis

Pancreatic cancer (PaCa) is the most lethal cancer, with a mortality rate close to the incidence rate. The overall 5-year survival rate is ~5% (29) and does not exceed 15–20% after surgery, the only curative treatment option, owing to local recurrence and metastatic spread. Furthermore, 80% of patients are inoperable at diagnosis (30). Though mortality rates for several common cancers decreased over the last decades (29), mortality rates increased for PaCa. Ductal PaCa, the most frequent subtype, is expected to be the second cancer-related cause of death after lung cancer by 2030 (31). The high mortality, due to early spread and radio- and chemotherapy resistance (32), is caused by a small population of CIC (33). Three additional contributing features are abundant stroma reactions, preferential dissemination along intrapancreatic nerves and pronounced immune deviation.

Unlike most tumors, PaCa cells may form only small islands within an abundant tumor stroma. The main cellular components are cancer-associated fibroblasts (CAF), predominantly deriving from pancreatic stellate cells (PSC) and inflammatory cells. The ECM consists of collagens, laminin (LN), fibronectin (FN), proteoglycans, and glycosaminoglycans and harbors soluble factors affecting tumor and host cells (34, 35). The PaCa stroma reaction, primarily promoting tumor growth, may hamper tumor progression in certain circumstances, indicating the need for further studies on composition and activities (36).

Perineural invasion (PNI) is most common in PaCa and an indicator of aggressive tumors and short survival (37). The pancreatic nerve fibers from the splanchnic nerves, dorsal root ganglion and the vagus become hyperinnervated and hypertrophic. The nervous system participates in all stages of PaCa development with neurotrophic factors and axon guidance genes overrepresentation or mutation. CAF and intrapancreatic immune cells also affect the intrapancreatic neurons (38), but intrapancreatic neurons and Schwann cells also signal toward the tumor cells (39, 40).

Finally, the PaCa stroma is replete with immune cells (41) that are almost exclusively immunosuppressive (42).

The steeply increasing incidence of most malignant PaCa demands intensifying efforts to clarify the underlying mechanisms. PaCa shares the consecutive steps of the metastatic cascade with most epithelial carcinoma, but also displays several peculiarities. Extensive stroma dysplasia, preferred routing of migrating tumor cells along intrapancreatic nerves and striking deviations toward immunosuppressive cells and factors account for the early spread. We will discuss those features, which quantitatively differentiate PaCa from the majority of epithelial cancer. Exosomes and PaCIC markers, both essentially contributing to the selective features, are introduced in advance.

## The Importance of Exosomes in Tumor Progression

Contact between single tumor cells detaching from the tumor mass and distinct non-transformed tissues and cells is an essential prerequisite for tumor progression. The crosstalk between metastasizing and non-metastasizing tumor cells and non-transformed cells mostly relies on message delivery by TEX and stroma cell-derived Exo.

Exo, small 40–100 nm vesicles delivered by live cells (43), disperse throughout the body, which allows for short and long-range communication (44). Exo expressing donor cell-derived components allows differentiating non-transformed cell-derived Exo from TEX (45). Exo components are function-competent (46) and highly effective intercellular communicators (47). Delivered messages modulate the ECM, non-metastasizing tumor cells (Non-CIC), and non-transformed cells including hematopoietic cells, EC, FB, nerves, and epithelial cells (48–51).

Exo biogenesis starts with early endosome (EE) formation. EE derive from the trans-Golgi network or internalized membrane microdomains (52). Distinct transport machineries guide EE toward multivesicular bodies (MVB) (53). Exo collect their cargo during inward budding of endosomes, called intraluminal vesicles (ILV), into MVB (54–56). LPAR1^1^, Alix/PDCD6IP^1^, and HSP70^1^ spur inward budding and SGPP1^1^ and diaglycerol^1^ are engaged in cargo sorting (57, 58). Loading are nonrandom processes. Protein loading is facilitated by mono-ubiquitination, acylation, myristoylation, higher order oligomerization, or sphingolipids forming ceramide (59–61). Annexin-II supports RNA sorting (62). Optionally, RNA becomes incorporated by affinity for the outer (cytoplasmic) raft-like MVB membrane (63). MiRNA loading is guided by a zip code in the 3′-UTR and coupling of RISC (RNA induced silencing complex) to specific EXO motifs binding to HNRNP (heterogeneous ribonucleoprotein) (55, 64). Selective lncRNA recruitment requires clarification (65, 66). ILV are guided toward the proteasome for degradation or toward the plasma membrane, supported by microtubules and Rab^1^ proteins (53, 67). SNARE^1^ and synaptogamins assist fusion with the plasma membrane (52, 53, 67). Released vesicles are called exosomes.

### Exosome Composition

The Exo membrane lipid bilayer contains integrated membrane proteins and lipid- or membrane protein-attached cytoskeletal and cytosolic signaling molecules. The Exo lipid envelop is composed of phosphatidylcholine, -ethanolamine, -inositol, prostaglandins, lysobisphosphatidic acid, sphingomyelin, cholesterol, GM3^1^/GRM6^1^, and PS^1^ (phosphatidylserine) (68), high PS levels differentiating Exo from microvesicles (69). Lipids are organized along with lipid carriers such as lipid-transporting FABP^1^. Lipid second messengers are involved in biogenesis, some requiring a link to lipids during ILV invagination, e.g., HSPA8 needs battenin (CLN3^1^) (70), formed by PLD2^1^ (71, 72). Ceramide triggers an ESCRT (endosomal sorting complex required for transport)-independent pathway of Exo biogenesis (73). Cholesterol enhances flotillin-2 positive Exo secretion (74). Lipid transporters such as ABCA3^1^ are also involved in Exo production (75). Thus, Exo carry bioactive lipids, related enzymes, fatty acid transporters, and lipid-related enzyme carriers and use lipids to fuse with target cells (76–78).

Exo protein characterization profited from improved mass spectrometry (MS) (79) to be followed by the exocharta database [http://exocarta.org/exosome_markers]. Exo also contain proteins engaged in biogenesis and vesicle transport and proteins actively recruited during ILV invagination. Tetraspanins are most strongly enriched constitutive Exo component (80–82). Other abundant proteins include adhesion molecules, proteases, major histocompatibility complex (MHC) molecules, HSP, TSG101^1^, ALIX, annexins, cytoskeleton proteins, metabolic enzymes, cytosolic signal transduction molecules, and ribosomal proteins (82, 83). Finally, PaCIC biomarkers are enriched in TEX (84–86). This is important as CIC drive the metastatic process (87–90), where Tspan8 (86, 91) and associated α6β4 (92–94), CD44v6 (95, 96), and linked cMET^1^ (96, 97), CD184/CXCR4^1^ that can associate with Tspan8 and CD44v6 (98–100), cldn7 (84, 101, 102), and associated EpCAM^1^ (84, 103, 104), LGR5/GPR49^1^ (105, 106) and CD133/PROM1^1^ (107, 108) are engaged in distinct steps of tumor progression.

Exo also contain mRNA. mRNA is produced and processed in the nucleus, transported to the cytoplasm and translated. These processes are controlled by proteins, mostly RNA binding proteins (RBP), which interact with mRNA (109) and together with additional regulatory RNA constitute the mRNA binding protein code (110–113). Notably, the activity of RBP varies depending on the cell's activation state. Thus, GAPDH^1^ binds the 3′UTR of IFNγ^1^ and represses translation in inactive, but not activated T-cells (114). RBP also account for localization and trafficking of RNA-protein complexes in cells (115, 116). Finally, the mRNA profile of Exo differs from that of cells (117), metabolic enzymes and proteins engaged in cell-cell and cell-matrix adhesion being frequently overrepresented (118–120), and possibly translated in Exo (121, 122).

Exo contain a large range of non-coding (nc)RNA. Most abundant are microRNA (miRNA) and lncRNA. miRNA host genes are transcribed by RNA polymerase II to form primary (pri)-miRNA. The Drosha^1^ endonuclease associates with the RBP DGCR8^1^ releasing the stemloop precursor from the flanking pri-miRNA transcript sequence. After export from the nucleus by exportin-5, Dicer in association with TRBP^1^ cleaves the precursor loop releasing the mature miRNA (123). One strand of this duplex RNA is integrated into the RISC complex, which contains argonaute linking the miRNA to target mRNA (124, 125). Importantly, miRNA with sequence motifs for sorting into ILV are efficiently transferred into Exo, some miRNA becoming undetectable in the donor cell (126, 127). Most miRNA bind to a large number of mRNA and most mRNA are targeted by more than one miRNA, providing hurtles for their potential therapeutic use, aggravated by the discussed mode whereby miRNA affect target cells (117, 128).

LncRNA, defined by a length of >200 bp, are abundantly recovered from Exo (129). LncRNA are involved in a large range of activities, including chromatin organization, gene transcription, mRNA turnover, protein translation, and macromolecular complex assembly (130–132). LncRNA can also be grouped according to functioning as signal, decoy, scaffold, guide, enhancer RNA, and short peptides (133). Signaling lncRNA regulate transcription (134). Decoy lncRNA sequesters regulatory factors including Tf, catalytic proteins, subunits of larger chromatin modifying complexes and miRNA (135). Scaffold lncRNA provide platforms for assembly of multiple-component complexes, e.g., ribonucleoprotein (RNP) complexes (136). Guide lncRNA drive RNP to specific target genes (137). Enhancer lncRNA (eRNA) influence the 3-dimensional organization of DNA, which may result from lncRNA being not released and tethering interacting proteins to enhancer regions (138). Finally, lncRNA can encode function-competent short peptides (139). Evidence for selective recruitment into Exo derives from enrichment of some lncRNA harboring seed regions for miRNA in Exo (140, 141). LncRNA recovery in Exo only recently receiving attention, important information on the multiple functions of lncRNA can be expected in the near future.

Exo contain mitochondrial, genomic, or retrotransposon double and single stranded DNA (142, 143). Without hints toward sorting and disputed functionality, a possible contribution of Exo DNA to tumor progression remains to be elaborated.

**Figure 1:**
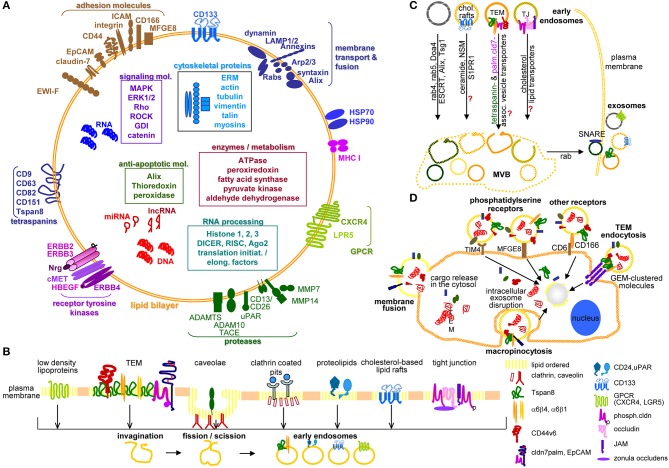
Exosome characterization, biogenesis, and targeting. **(A)** Exosomes are composed of a lipid bilayer, transmembrane protein and the cytoplasm containing proteins, mRNA, non-coding RNA like miRNA and lncRNA and DNA, where PaCIC-TEX, express the CIC markers Tspan8, integrin α6β1/α6β4, CD44v6, CD133, CXCR4, LRP5, EpCAM, and cldn7. Other transmembrane proteins are linked to Exo biogenesis. **(B)** Exo biogenesis starts with the invagination of membrane microdomains that are characterized by ordered lipids, like low-density lipoprotein, caveolae, clathrin-coated pits, cholesterol-based lipid rafts, and others. **(C)** After fission and scission of invaginated membrane domains, the EE are guided toward MVB, the traffic differs between the origins from distinct lipid-enriched domains. Most abundant is rab4, rab5, Doa4 promoted migration and invagination into MVB via the ESCRT system. Components of cholesterol-based lipid raft-, TJ-, or TEM-derived EE are not completely explored. Guidance from MVB to the plasma membrane involves rab proteins, phospholipase D, and SNARE. **(D)** The contact between Exo and target cells can proceed via fusion of the Exo membrane with the cell membrane, by macropinocytosis, receptor ligand binding such as phosphatidylserine binding to TIM4 or MFGE8 or CD166 binding to CD6 or may be facilitated by Exo membrane protein complexes binding to invagination-prone complexes as described for TEM binding to the TCR complex. Exo also bind to the ECM or matricellular proteins, CD44 and integrins being most frequently involved. Full name of proteins are listed in Table S1. In brief, cells use a variety of pathways for the generation of EE, the traffic toward MVB, the loading of ILV with proteins, coding and non-coding RNA and DNA. Exo may preferentially bind to and be taken up by receptor-ligand binding, uptake being facilitated by the engagement of protein complexes at both the Exo and the target cell.

Taken together, TEX are optimally furnished to drive all steps of the metastatic cascade using their lipid, protein and RNA armament, where PaCIC markers contribute to biogenesis (Tspan8), miRNA loading (CD44v6), and lipid transport (cldn7) (144, 145) (Figures 1A–C).

### Exosome Targeting and Uptake

Exosomes bind to the ECM and cells, using for both a similar appurtenance.

Exo binding mostly relies on surface receptor and adhesion molecules, such as tetraspanins, integrins, proteoglycans, and lectins, docking to appropriate ligands on the ECM and cells (146). Tetraspanin-associated adhesion molecules account for target-selective binding. Thus, Tspan8-α4 preferentially binds EC, whereas Tspan8-α6β4 preferentially binds FB (147, 148). Integrins, receptors for ECM proteins, also are involved in Tspan8-independent Exo binding (149), e.g., preventing α5β1-FN binding inhibits anchorage independent growth (150). ECM-binding proteins also guide Exo docking and uptake by recipient cells, demonstrated for β_1_, α_v_, β_3_, and α_L_ integrin chains and ICAM1^1^ (151). Recipient cell integrins contribute to Exo binding. PaCa-TEX preferentially bind ADGRE1^1^ and CD11b^1^ on Kupffer cells (152). Premetastatic niche formation relies on an integrin-dependent TEX tropism. (Tspan8)/β4 preferentially binds to lung (148, 153), αvβ5 preferentially to liver cells (153).

A second Exo docking system also is highly relevant (154). Exo proteoglycans bind to their receptors such as galectins, CD62E^1^, CD169/SIGLEC1^1^ (155, 156), and CD44 binds to hyaluronan (HA^1^) (157). Blocking Exo heparan sulfate proteoglycan (HSPG), the proteoglycan CD44 or the target cell ligands interferes with Exo binding *in vitro* and *in vivo* (157–160). PS binding TIM4^1^, TIM1^1^, TIM3^1^, GAS6^1^, MFGE8^1^, Stabilin1, ADGRB1^1^, and RAGE/AGER^1^ also contributes to Exo docking (146, 154, 161). Furthermore, we want to stress that protein complexes rather than individual molecules, many of which are abundantly expressed, likely account for the selectivity of Exo binding. This is well-demonstrated for tetraspanin complexes in glycolipid-enriched membrane domains (TEM), the multiple interactions between clustered proteins and target ligands strengthening and stabilizing docking (162). Finally, in view of the ongoing discussion on rapid Exo clearance *in vivo*, which could interfere with their therapeutic efficacy, an excellent report on CD47 binding to SIRPα^1^ preventing Exo clearance should be mentioned. Particularly in PaCa, oncogenic KRAS contributes to Exo uptake by yet undefined mechanisms such that long-term persisting Exo manipulated to target oncogenic KRAS is currently the most efficient therapeutics (163).

Exo uptake proceeds by Exo fusion (164, 165) or preferentially endocytosis, a process requiring actin modulation (166). Endocytosis occurs via phagocytosis, macropinocytosis, or clathrin-dependent lipid raft/caveolae endocytosis (167). Phagocytosis, facilitated by LAMP1^1^ and TIM4 proceeds by forming cup-like extensions, the tips fusing and becoming internalized (168, 169). Macropinocytosis relies on lamellipodia folding back and fusing with the plasma membrane. Dynamin, Na+H+ exchange, RAC1^1^, EGF, and SDF1^1^ are also engaged in uptake (170). Endocytosis via clathrin-coated pits, rafts, TEM or caveolae are most frequent (171, 172). In clathrin-dependent endocytosis, the membrane invagination becomes coated with clathrin. Clathrin-coated pits are released after scission by dynamin, dominant-negative forms of clathrin reducing Exo uptake (146). Ligand clustering in TEM also supports Exo uptake (162, 171) and a caveolin knockdown (kd) reduces exosome uptake (173, 174). Uptaken Exo are targeted to lysosomes for degradation. Exo content can directly modulate target cells or stimulate target cells' signaling cascades, transcription and silencing processes (175–177) (Figure 1D).

Exo/TEX binding and uptake drastically influence targets. In PaCa, TEX, but also PSC/CAF, immune cell and nerve Exo contribute to PaCa progression.

## Pancreatic Cancer-Initiating Cell Markers and the Metastatic Cascade

**Figure 2:**
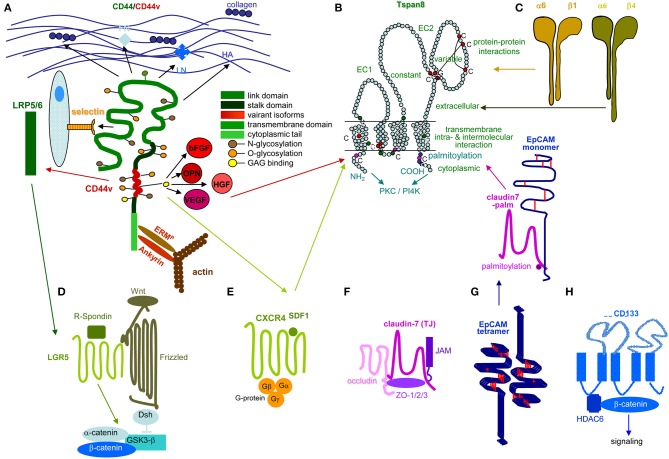
Prominent PaCIC markers. **(A)** The lead PaCIC marker is CD44v6, a type I transmembrane protein that contributes to the crosstalk with the ECM via its link domain and the HA binding site. It has binding sites for selectins and LRP5/6. The v6 exon product carries binding sites for several growth factors. The cytoplasmic tail has binding sites for ankyrin and ERM proteins including merlin, which promote cytoskeleton association and downstream signaling. **(B)** Tspan8 is a tetraspanin with a small and a large extracellular loop, the latter mostly accounts for protein-protein interactions. The four transmembrane regions account for intramolecular and intermolecular interactions. The cytoplasmic tail binds PKC and PI4K. Main activities rely on the association with a large range of proteins. Dominant are integrins, but also CD44v6 and an EpCAM-cldn7 complex. **(C)** Particularly α6β4 is known as a PaCIC marker. Similar to other integrins, it binds matrix proteins, particularly LN. It is a major component of hemidesmosomes anchoring epithelial cells in the basal membrane. Upon activation, it leaves the desmosome complex and associates preferentially with Tspan8. It differs from other integrins by a long cytoplasmic domain of the β4 chain, which promotes multiple signaling pathways. **(D)** LGR5 is a seven transmembrane protein located close to frizzled. Upon R-spondin binding, it contributes via Wnt activation to ß-catenin liberation. LGR5 activity is supported by CD44v6-associated LRP5/6. **(E)** CXCR4 is another seven transmembrane protein. This GPCR becomes activated by SDF1 binding. It predominantly signals via trimeric G-proteins. CD44 crosslinking via HA promotes CXCR4 recruitment and strengthens activation of downstream signaling cascades. Activated CXCR4 also associates with Tspan8 **(F)** Claudin7 is a 4 transmembrane protein, which can be integrated in TJ, where it associates with other claudins, JAM, and occludin and the cytoplasmic zonula occludens proteins. Cldn7 is also recovered outside of TJ. Upon palmitoylation, it associates via a direct protein-protein interaction in the transmembrane region with monomeric EpCAM. The cldn7-EpCAM complex is recruited into TEM and associates with Tspan8. **(G)** EpCAM is a type I transmembrane protein of many epithelial cells. It forms tetramers, which promote homophilic binding to EpCAM on neighboring cells. It is engaged in signal transduction, predominantly via the liberated cytoplasmic tail that acts as cotranscription factor. **(H)** CD133 is a five transmembrane protein located in cholesterol-rich membrane domains. It is associated with HDAC6 that stabilizes a ternary CD133-HDAC6-β-catenin complex and β-catenin target activation, which present one of the signaling cascades initiated via CD133. The seven most prominent PaCIC markers belong to distinct protein families and exert non-related functions. Five of these molecules can become recruited into TEM, where they associate via weak, non-protein-protein interactions with Tspan8. This significantly expands the range of activities of TEM and TEM-derived Exo. Of note, all seven CIC markers contribute via different routes to maintain stem cell features.

Metastasis depends on CIC. Stem cells are a rare cell population with the capacity for self-renewal and differentiation, which relies mostly on Tf activation, the nuclear equivalent remaining unaltered (178–180). This also accounts for CIC (181, 182), characterized by infrequent division (183, 184), longevity (185), drug and radiation resistance (186–192), and migratory activity (193–196). Since CIC depend on crosstalk with surrounding tissues (197, 198), we wondered whether the PaCIC biomarkers CD44v6 (Figure 2A), Tspan8 (Figure 2B) and associated α6β4 (Figure 2C), LGR5/GPR49 (Figure 2D), CXCR4/CD184 that associates with Tspan8 and CD44v6 (Figure 2E), cldn7 (Figure 2F), EpCAM and cld7-associated EpCAM (Figure 2G), and CD133 (Figure 2H) might provide hints toward feedback communications between PaCIC and the stroma.

### Tspan8 and the α6β4 Integrin

Tetraspanins are highly conserved 4-transmembrane proteins with a small and a large extracellular loop (199). The latter accounts for dimerization and association with non-tetraspanin molecules (200, 201). Prominent partners are integrins, proteases, cytoskeleton, and cytosolic signal transduction molecules (202–205). Intracellular, juxtamembrane cysteine palmitoylation supports tetraspanin-tetraspanin web formation, protects tetraspanins from lysosomal degradation and provides a link to cholesterol and gangliosides, tetraspanins mostly acting as molecular facilitators for associated molecules (206–209). As mentioned, Tspan8 contributes to Exo biogenesis (210) and is upregulated in PaCIC and -TEX (211–214).

Tspan8-promoted PaCa migration, invasion, and progression (215–220) relies on the recruitment of additional CIC markers. Tspan8 associates with CD44v6 (213), which recruits cMET and VEGFR2^1^ via CD44v6-bound HGF^1^ and VEGF^1^ (216, 221, 222), α6β1 and α6β4 (213, 223, 224), cldn7 and EpCAM (225–227). Some associations depend on the cells' activation state in particular α6β4 (228), a major hemidesmosome component in non-activated cells (229, 230). Upon association with Tspan8, integrins become activated and initiate downstream signaling (231, 232). The tight junction (TJ) component cldn7 (233, 234) only associates upon palmitoylation (234) and recruits EpCAM (235–238). Tspan8 also cooperates with proteases (239–241).

Tspan8/Tspan8-TEX engage in crosstalk with the tumor stroma and premetastatic niche tissue (210) and promote EC progenitor maturation and activation (147, 148, 242). The interaction with the ECM is initiated by Tspan8-associated integrins. Collagen crosslinking assists associated protease activation, which degrade collagen and LN (243). Tspan8-associated α6β4 binding to the LN332^1^-rich BM promotes tumor cell migration. Liberation of growth factors, chemokines and proteases deposited in the ECM supports tumor cell migration and distant organ settlement (157). TEX Tspan8-integrin and -protease complexes distinctly affect gene expression in different target cells. Tumor cells respond with vimentin, Snail^1^, and Slug^1^ expression. In FB proteases (ADAM17^1^, MMP14, TIMP1, and 2^1^) are mainly upregulated (240). Bone marrow cells (BMC) respond with TNFα^1^ upregulation and STAT4^1^ activation. Lymph node cells (LNC) upregulate TNFα, TGFβ, and FoxP3^1^ expression (240). TEX Tspan8-α4β1/α5β1 (147, 148) targeting EC/EC progenitors induce CXCL5^1^, MIF^1^, vWF^1^, and CCR1^1^ mRNA translation. The increase in mRNA after 1d−5d indicates induction of transcription (147). *In vivo*, EC/lymphatic EC respond with FGF2^1^, VEGFR1, VEGFR2, and VEGFR3 upregulation (244).

**Figure 3:**
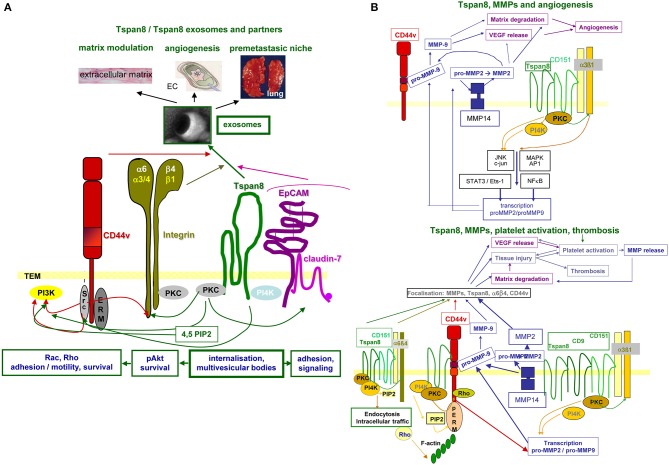
Tspan8 promoted tumor progression. **(A)** Tspan8 acts as a facilitator. This accounts for membrane bound Tspan8, where it strengthens CD44v6, integrin, and cldn7palm/EpCAM complex signaling activity via its association with PKC and PI4K. This also holds true for the Exo-recruited TEM complex described to modulate the ECM, to promote or inhibit angiogenesis and to contribute actively to premetastatic niche formation. **(B)** Tspan8 is associated with MMP14 and the association of Tspan8 with α6β1 promotes, besides other the transcription of MMP2 and MMP9. Upon proform activation, also assisted by the proximity to CD44v6, matrix proteins become degraded and VEGF is released. VEGF, in collaboration with collagen degradation products, promotes angiogenesis. In addition, a complex between Tspan8, CD44v6, α6β4, and MMP is found in focal contact. The matrix degradation promoted tissue injury contributes to platelet activation and thrombosis, where together with the release of VEGF a positive feedback loop is created further pushing platelet activation and thrombus formation. Full name of proteins are listed in Table S1. With the multitude of Tspan8 associating molecules, we only present one example building on the association with MMP, which strengthens angiogenesis and thrombus formation. However, it should at least be mentioned that Tspan8 also associates with TACE, which strongly affect e.g., the delivery of the NOTCH and the EpCAM ICD, both acting as cotranscription factors.

In brief, Tspan8 contributes to tumor progression at different levels of the metastatic cascade. Tspan8 is engaged in TEX biogenesis and binding/uptake and acts by clustering integrins, RTK, and proteases, which facilitate downstream signaling (Figure 3).

The α6β4 integrin was one of the first genes described to be metastasis-associated (245, 246). It is expressed in several normal epithelia, Schwann cells and EC, the β4 chain being characterized by a long cytoplasmic tail (245). A6β4 binds to LN in the BM facilitating adhesion through the formation of hemidesmosomes, nucleating the connection between LN and cytokeratin intermediate filaments (247). Upon stimulation, hemidesmosomes are dismantled allowing leading edge migration (248, 249). Hemidesmosome disassembly is accompanied by α6β4 forming a complex with MST1R/RON^1^, which interrupts its association with plectin (250). β4-linked activated ERBB2^1^ associates with src^1^, which initiates phosphorylation of the three components and signaling toward STAT3, which accounts for the breakdown of cell-cell junctions and initiation of invasion (251). Motility involves PI3K^1^ catalytic subunit beta activation, proceeding via α6β4 promoted IRS1 and−2^1^ phosphorylation (252), PI3K localization into lipid rafts or TEM (253, 254), or ERBB2/ERBB3 activation (255, 256). RAC1 activation strengthens the formation of F-actin-rich motility structures by the cooperation of α6β4 with RTK (257). α6β4-increased cAMP-specific phosphodiesterase activity decreases cAMP and activates RhoA (258). FAK^1^ regulates β4 tyrosine phosphorylation, which further promotes migration (259). Intravasation and extravasation are assisted by β4 cytoplasmic domain-dependent upregulation of VEGF enhancing transendothelial permeability (260). TEX Tspan8-α6β4 supports premetastatic niche preparations in the lung (92, 261).

β4 contributes to apoptosis resistance via tyrosine phosphorylation of the C-terminal segment of β4 by src family kinases downstream of RTK, but also by syndecan, which directly binds to the β4 cytoplasmic domain (262). Regardless of the initial signals, apoptosis resistance progresses by antiapoptotic PI3K pathway activation (263). TEX β4-vinculin complexes also cope with resistance toward a complex diterpene alkaloid, likely via plectin transfer by TEX (264).

Finally, α6β4 regulates transcription of invasion/metastasis-associated molecules by controlling promoter DNA demethylation. This was demonstrated for NFAT1^1^ (265), which assists autotoxin expression, a motility factor stimulating lipoproteinA production (266). Metastasin1/S100A4^1^ (267) spurs membrane ruffling via rhotekin (268), regulated through NFAT5 in conjunction with S100A4 promoter demethylation (269). S100A4 is also engaged in ERBB2 translation (270).

A6β4 is expressed on mature EC, a contribution to angiogenesis being disputed (271). Although reported to inhibit angiogenesis (148, 272, 273), α6β4 may be engaged in an early stage of angiogenesis (274) via stimulating VEGF translation and signaling (275). The β4 C-terminal domain is important for responding to FGF2 and VEGF (276) and arteriolar remodeling is defective in β4 knockout (ko) cells due to altered TGFβ signaling (271).

**Figure 4:**
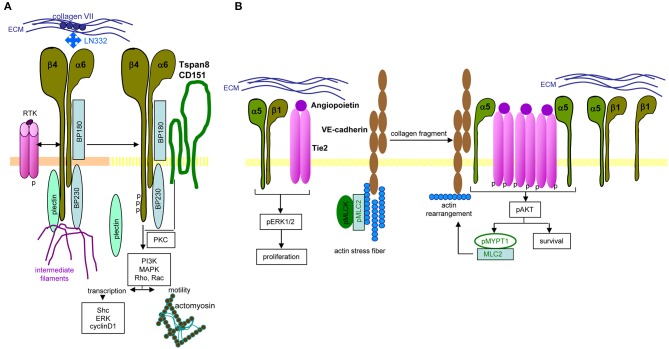
Distinct integrin signaling in PaCIC. **(A)** Hemidesomosome-integrated α6β4 is associated with BP160/320 and plectin, the complex being linked to intermediate filament. Upon contact with RTK, the β4 cytoplasmic tail becomes phosphorylated, plectin is released from the complex and phosphorylated β4, supported by Tspan8-associated PKC promotes PI3K, MAPK, Rho, and RAC activation. Besides initiating transcription, the complex assists the association with actomyosin and motility. **(B)** Instead, when α5β1 associates with angiopoietin-activated Tie2, proliferation is initiated via ERK phosphorylation. In the presence of VE-cadherin, linked to actin stress fibers, pMLCK, and pMLC2 collagen fragments initiate actin rearrangement that promotes dissociation of the α5 from the β1 chain, which enclose phosphorylated Tie2. The phosphorylated Tie2 promotes Akt phosphorylation, which supports MYPT1 phosphorylation and MLC2 association that evoke actin rearrangement. Full name of proteins are listed in Table S1. In brief, only parts of integrin-mediated activities are affected by the association with Tspan8. Notably, the same stimulus distinctly affects integrin activation depending on the α or β chain of the integrin.

Long-known as metastasis-associated, molecular pathways of α6β4 are not fully unraveled. Central are the signaling domain of the β4 tail and the dislodgement from hemidesmosomes. In PaCIC/-TEX, we consider the linkage to Tspan8 as a central coordinator (Figure 4).

### CD44v6 and CD44v6-Associated Receptor Tyrosine Kinases

CD44v6, the alternatively spliced isoform of the adhesion molecule CD44 is a PaCIC marker involved in several steps of the metastatic cascade (277, 278). CD44, a type I transmembrane glycoprotein, varies in size by *N*- and *O*-glycosylation and insertion of alternatively spliced exon products between exons 5 and 6 of the CD44 standard isoform (CD44s) (279–281). CD44 belongs to the cartilage link protein family (282), the globular structure being stabilized by conserved cysteines (283). After the globular domain a heavily glycosylated stalk-like structure has putative proteolytic cleavage sites (284) and contains the variable exon products (285). The transmembrane region facilitates oligomerisation and recruitment into TEM, important for the interaction between CD44 and extracellular ligands and other transmembrane and cytoplasmic molecules (286). The cytoplasmic tail binds signaling and cytoskeletal linker proteins (287, 288). Most CD44s activities are maintained by CD44v.

CD44 has multiple ligands, which contribute to tumor progression. The link domain binds collagen, LN, FN, E-, and L-selectin (289, 290). CD44 has binding sites for glycosaminoglycans (GAG) and is the major HA receptor that binds to a basic motif outside the link domain (291–293). CD44v6 binds HGF, VEGF, and osteopontin (294–296). These associations are of central importance for its lateral associations with RTK. HGF binding brings CD44v6 into proximity with cMET and expedites cMET activation, which requires interaction between the CD44 cytoplasmic tail and ERM (ezrin, radixin, moesin) proteins for Ras^1^-MAPK^1^ pathway activation (297). CD44v6-ECM binding also contributes to cMET transcription (298). Lateral association-initiated signal transduction also accounts for IGFR1^1^ and PDGFR^1^ (299). The HA crosslinking-initiated CD44 association with CXCR4 promotes SDF1 binding (300). The association with the low-density lipoprotein (LDL^1^) receptor-related LRP6^1^ strengthened activation of the EMT-related Wnt^1^ signaling pathway (301). Cytoplasmic tail-bound ankyrin contacts with spectrin support HA-dependent adhesion and motility (287). ERM proteins regulate migration, cell shape, and protein resorting (302, 303). The N-terminus of activated ERM proteins binds CD44, the C-terminus F-actin (304). Cytoskeletal linker protein binding expands the range of CD44-mediated downstream signaling pathways (303, 305), which can also proceed directly from TEM-located CD44v (306–308) or associated non-RTK (309, 310). The CD44/CD44v6-associated membrane-bound proteases MMP14 and Hyal2^1^ (311) support tumor cell migration through matrix degradation and remodeling (312). CD44 contributes to drug resistance (313) by associating with ABC^1^ transporters (314, 315) and additional antiapoptotic proteins (316, 317). Last, not least, the CD44 cytoplasmic tail (CD44ICD) moves toward the nucleus functioning as a cotranscription factor (318). Alternatively, the CD44v6 cytoplasmic tail can affect transcription by activation of signal-transducing complexes. With regard to the metastatic cascade, CD44v6 was described to directly or indirectly activate Tspan8, MMP9, MDR1^1^, and NOTCH1^1^ transcription (221, 319–321). Finally, CD44v6, but not CD44s, is engaged in loading ILV with miRNA (159, 322), which might rely on its association with Dicer (322) and contributes to tumor progression (323).

**Figure 5:**
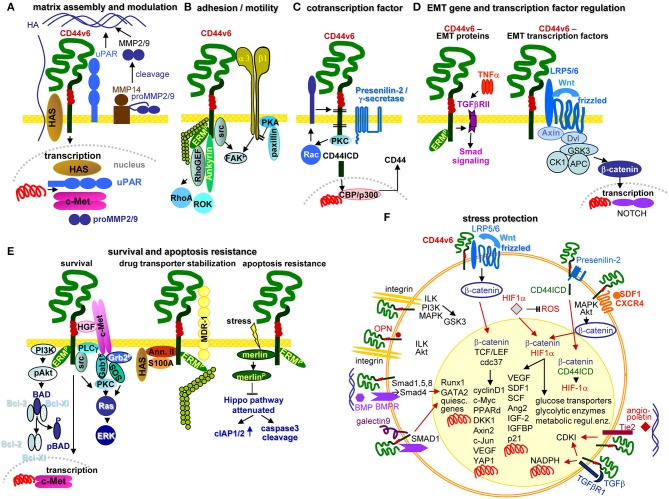
Multifaceted activities of CD44v6 in PaCIC. **(A)** Upon HA crosslinking, CD44v6 initiates HAS, uPAR, MMP2, and MMP9 transcriptions, which promote HA assembly and matrix remodeling, where MMP14 contributes to proMMP2 and MMP9 cleavages. **(B)** CD44v6 can associate with α3β1 such that both molecules jointly contribute to FAK activation and motility. **(C)** CD44v6 can be cleaved by TACE and subsequently by the presenilin2 complex. The CD44ICD acts as a cotranscription factor, which together with CBP/p300 promotes CD44 transcription **(D)** By TNFα associating with TGFβRII, EMT protein expression is supported via Smad signaling. The association of CD44v6 with LRP5/6 supports Wnt/frizzled activation such that β-catenin leaves the suppressive complex and acts as cotranscription factor in NOTCH transcription. **(E)** There are several pathways whereby CD44v6 strengthens PaCIC survival and apoptosis resistance. cMET comes into proximity of CD44v6 via CD44v6-bound HGF. This initiates activation of the PI3K/Akt anti-apoptotic and of the Ras-ERK pathways. In addition, CD44v6 supports cMET transcription. A complex of CD44v6 with HAS, Annexin II, S100A, and activated ERM stabilizes MDR1 expression, which contributes to drug efflux. Finally, stress induces the association with and dephosphorylation of merlin, which attenuates the HIPPO pathway with upregulation of cIAP1/2 and caspace3 cleavage. **(F)** Some of the multiple activities of CD44v6 in stress protection via affecting the cells metabolism are summarized indicating whether altered metabolism is promoted by signaling cascades in the cytosol or depends on transcriptional activation (red arrows). The latter accounts particularly for β-catenin-TCF/LEF, β-catenin-HIF1α, and β-catenin-CD44ICD complexes, but also for the cooperation of CD44v6 with Tie2, TGFβR1, galectin 9, and BMPR, which affect transcription of a large range of distinct genes. Full name of proteins are listed in Table S1. CD44v6 is engaged in most steps of the metastatic cascade. The strongest impacts are seen in terms of survival, EMT induction and metabolic changes that guarantee unimpaired survival under hypoxic and poor nutrient conditions.

In brief, CD44v6 engages in EMT induction by supporting Wnt signaling and Nanog and Notch activation (324–326). It contributes to intravasation through binding and degradation via associated proteases. It supports extravasation by selectin binding to EC, allowing crawling toward EC-EC gaps. It assists tumor stroma formation and premetastatic niche preparation by HA, matrix-remodeling enzyme, cytokine, and chemokine provision (91, 327). Recruiting miRNA into ILV expands the range of TEX activities (322). A few of the many CD44v6 activities in tumor progression are shown in the accompanying figure (Figure 5).

### CXCR4 and Its Association With Tspan8 and CD44v6

CXCR4 has been tied to tumor progression and poor prognosis (328, 329) and expression of its ligand SDF1 correlates with poor survival (97).

CXCR4 is expressed in BMC/-precursors, lymphocytes, resident macrophages (Mϕ), EC precursors, FB, and CIC. CXCR4 is a seven transmembrane GPCR (330), transcription increasing in response to several signaling molecules such as cyclic AMP, some cytokines including TGFβ and the growth factors FGF2 and VEGF (331). Upon ligand binding, CXCR4 undergoes a conformational change activating the intracellular trimeric G protein leading to the Gαi dissociation, which stimulates src, Ras/Raf^1^/MAPK (332) and PI3K pathways (331, 333). Gβγ triggers PLC, which catalyzes PIP2 into IP3 and DAG leading to Ca++ mobilization and PKC^1^ and MAPK activation (334). CXCR4 also triggers a G-protein-independent pathway (335) promoting recruitment of GRK2^1^ that phosphorylates the C-terminus resulting in β-arrestin association. CXCR4 thereby uncouples from G proteins and becomes internalized (336, 337). GRK2 is supported by PKC, PKA, and src (338). β-arrestin serves as a scaffold for downstream signaling promoting ERK/MAPK1 and p38/MAPK14 activation (339). Proper folding depends on HSP90, a chaperone for members of the CXCR4 phosphorylation cascade (340). Colocalization of these complexes in cholesterol-enriched lipid rafts (341) facilitates signal transfer (342).

CXCR4 contributes to tumor progression at multiple levels. CXCR4 sustains proliferation through direct activation of MAPK, PI3K, Wnt, and HH^1^ signaling (343), where HH additionally induces SDF1 expression in the tumor surrounding (344) and activation of the intrinsic anti-apoptotic pathway via ERK and Akt^1^ (344, 345). CXCR4 assists invasion, HH signaling being associated with EMT and loss of adhesion (344). SDF1 increases MMP2, MMP9, and urokinase expression (346, 347). Particularly in PaCa, CXCR4 expression is linked to a subpopulation of migrating, metastasis-promoting PaCIC (348) that is highly chemotherapy resistant (349–351).

**Figure 6:**
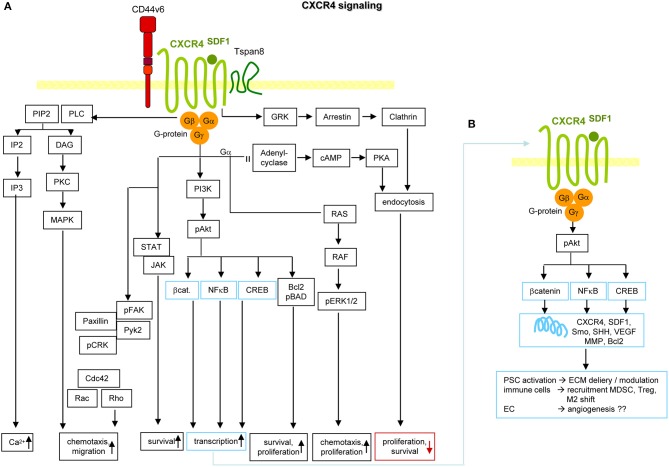
CXCR4 and PaCIC survival and motility. **(A)** CXCR4 is a G-protein coupled receptor (GPCR) that in PaCa is mostly recovered in association with CD44v6 and/or Tspan8. Activation is initiated by binding of its ligand SDF1. Signals are transferred via the G protein subunits, which promote Ca2+ influx, and either via MAPK or Rho chemotaxis and migration. Chemotaxis and proliferation can also proceed via the Gα, Ras, Raf, pERK1,2 activation route. Activation of PI3K/Akt, Bcl2/pBAD promotes proliferation and survival. The latter is also supported by activation of the STAT-Jak pathway. PI3K/Akt can also initiate activation of transcription factors. Independent of the trimeric G-protein complex, CXCR4 associates with GRK, arrestin and clathrin. The complex becomes internalized, which is accompanied by reduced proliferation and survival. **(B)** Activation of β-catenin, NFκB and CREB supports transcription of CXCR4, SDF1, Smo, SHH, VEGF, MMP, and Bcl2. These genes are important in PSC activation, recruitment of immunosuppressive MDSC and Treg and the shift of M1 to M2 and in supporting angiogenesis, which may not be dominating in PaCa. Full name of proteins are listed in Table S1. It should be noted that the dominant activity of CXCR4 in promoting chemotaxis and motility covers only one, not essentially dominating feature in PaCIC.

The involvement of CXCR4 in tumor progression is not restricted to tumor cells. EC respond to HIF1α^1^ with CXCR4 upregulation (352). The SDF1-CXCR4 axis enhances VEGF and MMP production through ERK and Akt signaling (353), which promotes EC migration and capillary tube formation (354). Activated PSC (aPSC) promote SDF1 secretion, which binds to EC CXCR4 and is supported by PAUF^1^. SDF1 together with VEGFC also attracts lymphatic EC (354). Furthermore, tumor stroma cell-secreted SDF1 assists CXCR4 activation in tumor cells and CXCR4-induced HH expression stimulates CAF recruitment (344). By stimulating IL6^1^ production, CXCR4 assists TAM recruitment (343) and mast cell recruitment and activation. Mast cells release IL13, which activates PSC, further promoting tumor growth (355). Other CXCR4-recruited immune cells force CXCR4 expression via IFNγ creating a positive feedback loop (356). The link between high CXCR4 expression and bone metastases relies on circulating tumor cells passing through the bone vessels, hematopoietic and mesenchymal progenitors highly expressing SDF1 (357). Another CXCR4 ligand is SDF1-associated HMGB1^1^, which is also a ligand for AGER (358) and TLR2, 4, and 9^1^ (359, 360). SDF1/HMGB1 complex binding to CXCR4 promotes inflammatory cell recruitment (361) (Figure 6).

In 2007, a first series of reviews pointed out the special role of CXCR4 in subpopulations of migrating/metastasizing CIC (348, 362, 363). Great progress over the last decade extended original findings toward the involvement of tumor stroma and EC. Although the extent of CXCR4 heterocomplex engagement in leukocyte recruitment awaits further exploration (364), based on promising results, great efforts are taken toward therapeutic translation (100, 365, 366).

### Claudin7 and EpCAM

Claudins, including cldn7, are a four-pass proteins, which are the central TJ components (232, 367). TJ are found in epithelial and endothelial cells, cldn7 expression being particularly high in the gastrointestinal tract and lymphatic vessels (368). TJ, composed of the transmembrane proteins occludin, JAM and cldn, linked to zonula occludens proteins (ZO^1^), are located in the most apical lateral region of cell-cell contact sites (367). The transmembrane proteins are laterally linked via claudins, and tightly associate with TJ on opposing cells (369). TJ seal the organism from the outside and are involved in paracellular transport (370). The latter is determined by the polarity of the β-sheet of the extracellular loops of cldn, which differs between individual cldn and is adjusted to selective organs' demands (371). Both barrier and channel functions of TJ-integrated cldn are vital. Cldn7ko mice die within 10 days after birth due to gut destruction that might rely on a missing association with integrins and strong MMP3 upregulation or on enhanced paracellular influx of colonic inflammation-inducing bacterial products (372, 373). Apart from sealing and paracellular transport (370, 371, 374–376), few reports explore cldn-Exo/TEX activities. It was recently realized that a comparably large amount of continuously remodeled TJ components is recovered insight the cell and at distinct membrane locations (377–379). TJ remodeling rests on claudins being PKA, PKC, and MLCK^1^ targets, cldn phosphorylation prohibiting TJ integration (380–385). Furthermore, TJ formation depends on sphingomyelin with long-chain fatty acids and cholesterol enrichment in membrane subdomains, cholesterol depletion affecting cldn integration and abolishing TJ formation (386). Finally, cldn7 is also located in the plasma membrane outside of TJ. Cldn7 palmitoylation is a precondition for partitioning into TEM, where palmitoylated cldn7 associates with EpCAM and tetraspanins (234, 387).

Internalized, TJ-derived cldn can be degraded, recycle or integrate into EE and, after passage through MVB, into Exo. In fact, TEM-located, palmitoylated and EpCAM-associated cldn7 is exclusively recovered from apical plasma membrane derived TEX (388, 389). In organoids, a second population of cldn7+/EpCAM- TEX is delivered at the basal membrane (389), which might derive from internalized TJ, facilitated by the high cholesterol content. Intracellular vesicle traffic remains to be defined (378). Alternatively, Exo-recruitment during biogenesis is not excluded (390) and would be consistent with pronounced coimmunoprecipitation of cldn7 with Golgi-endoplasmic reticulum (ER) transporters (391).

**Figure 7:**
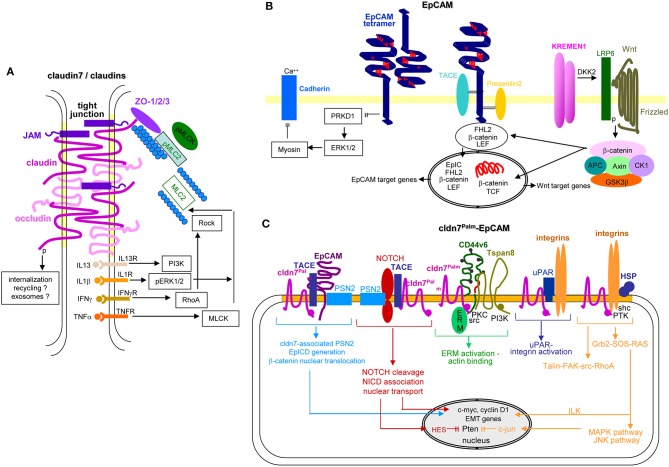
EpCAM, claudin7 and their cooperation in PaCa. **(A)** In tight junctions, cldn7 is associated with additional cldns, occludin, JAM, and ZO1, 2, and 3, the latter being associated with pMIC2 and the cytoskeleton. Upon stimulation by several cytokine receptors, PI3K, ERK, RhoA/Rock, and MLCK promote MLC2 dephosphorylation, which promotes reorganization of the cytoskeleton with consequences on the cldn associated transporter activity. A stress response provokes internalization of the TJ complex. It is suggested that the internalized complex may be partly digested, recycle and become integrated into Exo. **(B)** EpCAM form tetramers, which with low affinity bind to EpCAM tetramers on neighboring cells and concomitantly prevent PRKD1 activation that leads to ERK1/2 and myosin activation, which inhibits Ca++-dependent Cadherin adhesion. Alternatively, EpCAM becomes cleaved by TACE and subsequently by PSN2. The cotranscription factor EpICD becomes supported by LEF and ß-catenin that might derive from Kremen1-DKK2-LRP6 promoted Wnt-Frizzled activation. This transcription factor complex mostly supports EpCAM and Wnt target gene expression. **(C)** Palmitoylated cld7 associates with monomeric EpCAM. As cldn7 is associated with PSN2, EpICD generation is augmented. Palmitoylated cldn7 may also contribute to NICD generation that acts as cotranscription factor. In association with CD44v6 and Tspan8, cldn7palm associates with ERM and contributes to ERM activation and actin binding. In association with uPAR and integrins it promotes both uPAR and integrin activation. Finally, a cldn7Palm-integrin-HSP complex assists talin-FAK-src-RhoA activation and by activation of the Grb2-SOS-RAS pathway ILK and the MAPK-JNK pathway. EpICD, NICD, and ILK contribute to c-myc, cyclinD1 and EMT gene transcription; NICD via HES and c-jun interfere with Pten transcription. Full name of proteins are listed in Table S1. Thus, TJ cldn7 is important particularly in lipid transport and cytoskeleton organization, EpCAM by promoting oncogenes and EMT genes, which also accounts for cldn7Palm-associated EpCAM. Cldn7Palm additionally contributes to Pten silencing.

Pinpointing the activity of cldn7 in the metastatic cascade is difficult. Palmitoylated, EpCAM-associated cldn7 might favor signaling by supporting EpCAM cleavage and EPICD cotranscription factor activity in EMT. However, it is hard to demarcate from support by other TEM-located CIC markers. TJ-integrated and phosphorylated cldn7 is associated with a wide range of transporters, which likely impacts altered metabolism and signal transduction of CIC (Figures 7A,C). These options await untangling exploration.

The epithelial cell adhesion molecule EpCAM, overexpressed in many epithelial cancer, serves as diagnostic and therapeutic target (392). EpCAM mediates homophilic cell-cell adhesion (393, 394) and fulfills condition-dependent distinct functions (395). An initial, straight-forward explanation that oncogenic and tumor progression supporting EpCAM activities rest on interfering with E-cadherin-mediated adhesion required revisiting, when it was realized that EpCAM can be cleaved by TACE and subsequently presenilin1, which generates EpICD (396). EpICD functions together with TCF/LEF^1^ as a cotranscription factor for MYC, cyclinA/E, Oct4^1^, and Nanog amongst others (397, 398). EpICD is also engaged in hypermethylation and activation of BMP^1^ promoters (399) and can promote EMT through increased Slug and PTEN/Akt/mTOR^1^ signaling pathway activation (400) and engagement in Wnt signaling. PKC downregulation and MMP7 upregulation backs EpCAM-promoted motility (401–406). Indicating its regulatory effect on another major pathway, EpCAM silencing reduces Ras/Raf/ERK signaling (407). However, EpCAM expression is transiently downregulated during EMT (401, 408, 409), which could argue for EpCAM prohibiting tumor cell dissemination (410, 411). Nonetheless, strong overexpression on embryonic SC endorses a contribution to pluripotency maintenance (412, 413).

EpCAM expression is epigenetically regulated. High EpCAM expression correlates with hypomethylation of a fragment of exon 1 and the proximal promoter, lack of EpCAM expression correlates with methylation at a proposed Sp1 binding site (414, 415). Furthermore, activating histone modifications acH4^1^, acH3^1^, and H3K4me3^1^ correlate and repressive histone modifications H3K9me3^1^ and H3K27me3^1^ inversely correlate with EpCAM expression (413, 416, 417). Additionally, EpCAM regulation by ncRNA might be relevant to the crosstalk between TEX and targets. LncRNA LINC00152 activates mTOR through binding to the EpCAM promoter region (418). Furthermore, miR-150, miR-155, miR-181, and miR-223 expression is increased in EpCAM+ hepatocellular carcinoma (HCC). MiR-155 contributes to EpCAM-promoted migration and invasion (419) and miR-29b to proliferation and inhibition of liver progenitor cell differentiation (418). Since miR-16-5p, miR-23a-3p, miR-23b-3p, miR-27a-3p, miR-27b-3p, miR-30b-5p, miR-30c-5p, and miR-222-3p are high in EpCAM+ colorectal cancer (CoCa) TEX, an EpCAM-dependent recruitment is discussed (420).

In brief, possibly due to abundant expression in most epithelial cells and upregulated expression in many primary tumors, the CIC features of EpCAM are more difficult to define than originally expected. Notwithstanding, EpICD contributes to the metastatic process by acting as a cotranscription factor. We personally interpret the transient downregulation during EMT as evidence for EpCAM not contributing to intravasation, intravascular traffic or extravasation. Expression during settlement of migrating tumor cells in distant organs could indicate a share in premetastatic niche preparation (Figures 7B,C). An interpretation of EpCAM regulation by lncRNA and miRNA might be premature.

### LGR5

The leucin-rich repeat containing GPCR-5 (LGR5^1^) is a Rhodopsin GPCR, expressed in adult SC and best explored in intestinal SC and CIC (421). Secreted Wnt proteins interact with the Wnt receptor complex consisting of Frizzled and LPR5/6. Wnt binding sustains dissolving the downstream destruction complex and liberated β-catenin acts together with TCF/LEF as Tf (422). LGR5 is one of the targets of TCF4^1^ (423), which regulates Wnt signaling. In the absence of Wnt, Frizzled, LPR5/6 and the RING-type E3 ubiquitin ligases RNF43^1^ and ZNRF3^1^ form a complex, which promotes Frizzled ubiquitination and degradation. Upon soluble R-spondin binding to LGR5, RNF43 becomes phosphorylated and sequestered generating a more stable complex between R-spondin, LRP5/6, and Wnt-Frizzled, which promotes β-catenin liberation (424, 425). This suggests LGR5 elimination hampering tumor progression. LGR5 elimination transiently retarded local tumor growth, possibly reflecting CIC plasticity, where differentiated cells can revert to LGR5+ CIC. Instead, metastatic growth was enduringly inhibited (426, 427).

**Figure 8:**
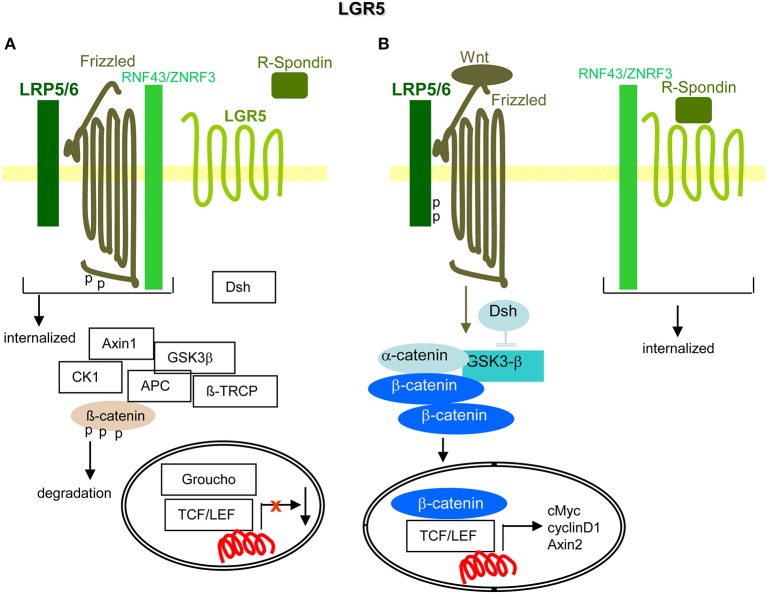
LGR5 and the contribution to PaCIC maintenance. **(A)** The leucine-rich repeat-containing GPCR is engaged in Wnt signaling. Its ligand is R-Spondin. In the absence of R-Spondin, the transmembrane E ligases RNF43/ZNRF3 associate with Frizzled and LRP5/6 that leads to Frizzled phosphorylation and internalization of the complex. **(B)** However, in the presence of R-Spondin, RNF43/ZNRF3 is recruited toward LGR5 such that Wnt can bind to Frizzled and LRP5/6 becomes phosphorylated. Dsh blocks GSK3-β and β-catenin is liberated to move to the nucleus, where it together with TCF/LEF promotes cMyc, cyclinD1, and Axin1 transcription. Full name of proteins are listed in Table S1. The upregulated expression of LGR5 in PaCIC suggests its engagement in PaCIC maintenance.

Briefly, by regulating Wnt signaling, LGR5 is important for CIC maintenance and thereby tumor progression (Figure 8).

### CD133

CD133 (Prominin1) is a hematopoietic SC and CIC marker in many malignancies (428, 429), high expression being associated with poor prognosis (430). CD133 is a 5-transmembrane molecule in protruding membrane subdomains, where it interacts with cholesterol-based lipid rafts (428, 431). CD133 contributes to cell polarity, cell-cell and cell-ECM interactions (432) and signaling cascade activation (433). Expression is enhanced by binding to HDAC6^1^ that stabilizes β-catenin in a ternary CD133-HDAC6-β-catenin complex promoting β-catenin target activation. Loss of CD133 is accompanied by reduced SLUG, LAMC1^1^, and MMP7 expression and a shift toward MET (434). CD133 activity is regulated by the tyrosine phosphatase receptor PTPRK^1^, which dephosphorylates tyrosines 828 and 852. Low PTPRK expression in patients with cancer is associated with pronounced AKT activation and poor prognosis (435).

CD133 interferes with CIC differentiation by suppressing NTRK2 via p38MAPK and PI3K signaling (436). A CD133kd is also accompanied by a strong decrease in invasion and TIMP2 expression, the pathway remaining to be explored (437). CD133 affects migration via Akt or src activation and FAK phosphorylation (438, 439). A suggested engagement in drug resistance might proceed via CD133 directly interacting with PI3K-p85, resulting in multidrug resistance (440). Finally, neighboring cells support CD133 activities, e.g., EC secrete a soluble form of Jagged1^1^ promoting Notch activation (441).

According to the location in internalization-prone rafts, CD133 is recovered in Exo/TEX (442–444). CD133 intracellular traffic follows an ESCRT-independent pathway and requires ceramide, neutral sphingomyelinases and the sphingosine-1 phosphate receptor S1PR1^1^, confirmed by reduced MVB formation upon expulsion of S1PR1 by α-synuclein^1^ (445, 446). The expected CD133-TEX contribution to intercellular communication requires exploration (107). However, endosomal CD133 at the pericentrosomal region captures GABARAP^1^, an initiator of autophagy. This prevents GABARAP from mediating ULK1^1^ activation and autophagy, whereby pericentrosomal CD133 sustains CIC undifferentiated state maintenance (447).

CD133 shares with several metastasis-associated markers the recovery in SC and CIC. It is engaged in CIC maintenance, Wnt/β-catenin signaling and contributes to migration and invasion, molecular mechanisms being not fully elucidated.

### CIC Markers, Stemness, and EMT

Before summarizing the importance of PaCIC markers in tumor progression, we need commending on the linkage between CIC and EMT. Partial activation of the embryonic EMT program was considered a central feature of CIC and a prerequisite for metastasis formation (5). This was recently questioned for PaCa, where the EMT-related Tf Snail and Twist do not contribute to PaCa metastasis, but promote proliferation (448). On the other hand Notch2 and its ligand Jagged-1 are highly upregulated in drug-resistant PaCa cells and a NOTCH2 kd is associated with a partial reversion of the EMT phenotype with decreased vimentin, ZEB1, Slug, Snail, and NFκB expression (449). A more recent publication, describing ZEB1 being essential for PaCa progression, offers a plausible explanation, proposing context-dependent complementary subfunctions of distinct EMT-related Tf (450). Thus, the suggestions of CIC stemness and (partial) EMT requirement in supporting tumor progression, are not yet unambiguously answered (5). Taking the frequently unimpaired growth of the primary tumor mass and of established metastases after therapeutic trials to deplete CIC markers and/or selected Tf, we expect that both stemness markers and partial EMT greatly facilitate tumor progression.

Despite remaining open questions, we want to close this chapter with a personal experience, dating back to 1978, where a local tumor and ascites of a spontaneously arising PaCa were isolated from a rat (451). After subcutaneous transfer, rats receiving local tumor tissue developed local tumors, but not metastases. Rats receiving ascites did not develop a local tumor, but metastases in draining and distant lymph nodes and became moribund due to thousands of miliary lung metastases (452). The local tumor does not, the metastasizing tumor expresses all previously listed PaCIC markers (453). While overexpression of CD44v6, Tspan8, β4, EpCAM, and cld7 supported selective metastasis-associated features, but not the full-fledged metastatic profile (242, 454–457), a kd of each of these markers was accompanied by loss or strongly reduced metastasis formation (240, 388, 458, 459). CIC being unknown at that time, our “blind” studies may convincingly demonstrate the strong impact of CIC markers in tumor progression, their interdependent activities, and importantly, the requirement for a tumor-host crosstalk, the topic of the following chapters.

## Stroma Dysplasia in Pancreatic Cancer

PaCa is characterized by an exuberant desmoplastic stroma reaction (DR) that may occupy far more space than the tumor cells, which form small nodules embedded in the dense DR (460). The DR is composed of ECM proteins, PSC, FB, EC, immune cells, and neurons (461).

**Figure 9:**
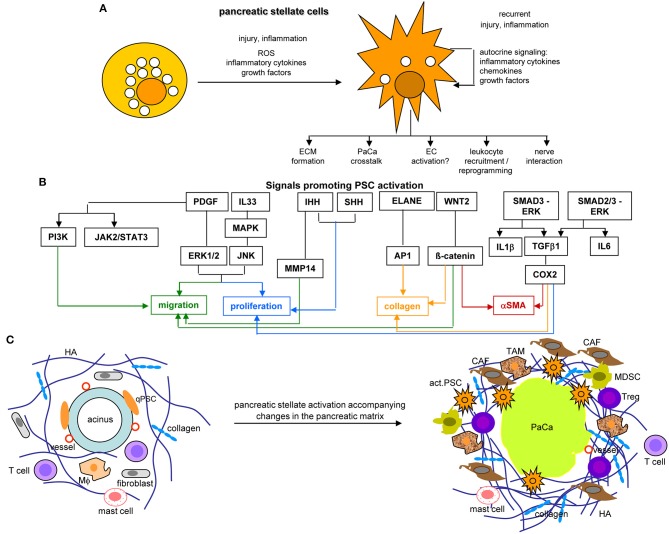
The core position of pancreatic stellate cells in the dysplastic stroma reaction in PaCa. **(A)** PSC abundantly contain lipid droplets and lay close to the acinar cells in the healthy pancreas. They become activated by injury or inflammation, with a contribution of inflammatory cytokines, growth factors and ROS. Recurrent injury promotes autokrine signaling with further provision of growth factors, inflammatory cytokines, and chemokines. They partly loose the lipid droplets and become dispersed throughout the pancreatic stroma, where they affect the ECM, PaCa cells, leukocytes, and nerves. **(B)** Main factors contributing to PSC activation are PDGF and IL33 that assist proliferation and migration, Wnt2-β-catenin and IHH-MMP14 also contribute to the migratory phenotype and IHH-/SHH-Cox2 to proliferation. ELANE-AP1, Wnt2-β-catenin, and Smad3-ERK-TGFß1-Cox2 support collagen secretion, the latter two also support αSMA expression. **(C)** PSC activation is accompanied by the generation of a very dense ECM rich in HA and collagen, the recruitment of CAF, TAM, MDSC, and Treg, but a paucity of T cells in the dense ECM. Finally, they are engaged in a most intense crosstalk with the PaCa cells. Full name of proteins are listed in Table S1. PSC become activated at an early stage of PaCa initiation. Signals promoting PSC activation contribute to PSC collagen and αSMA expression, proliferation, and migration. aPSC are supposed to account for the ECM formation, to crosstalk with the tumor cells, to recruit and reprogram of leukocytes and to interact with the intrapancreatic nerves, some of these activities are detailed in the following figures.

PSC, quiescent in the healthy pancreas, are located in the basolateral region of acinar cells (462, 463). They are characterized by GFAP^1^, desmin, vimentin, nestin, NGF, and NCAM^1^ (464). During pancreatic injury, PSC develop a myofibroblast phenotype expressing αSMA^1^, actively proliferate and migrate. Activation of PSC is promoted by TGFβ, HGF, FGF, EGF, and sHH^1^ (465) (Figures 9A,B).

Activated PSC (aPSC) modulate the tumor matrix. They secrete ECM proteins including collagen I, III, and IV, FN and LN (464). Matrix deposition is supported by epithelial cell secreted SERPINE2^1^, which activates PSC resulting in enlarged ECM protein deposits (466). PSC secrete MMP2, MMP9, MMP13, TIMP1, and TIMP2, which account for matrix modulation (467–470). aPSC also affect immune cells. They express TLR2-5, required for non-adaptive immune cell activation (471), but also TLR9, which is protumorigenic via CCL11. CCL11 recruits regulatory T-cells (Treg) and promotes myeloid-derived suppressor cell (MDSC) proliferation (472). aPSC express MHCII and present tumor antigen peptides (473). However, in the absence of costimulatory signals MHC II presentation is not sufficient for helper T-cells (Th) activation (474). Further supporting immunosuppression, aPSC express high level of CXCL10/IP10^1^, which correlates with a Treg increase and reduced CTL (cytotoxic T lymphocyte) and NK (natural killer cell) activity (475). aPSC also express T-cell apoptosis-inducing GAL1 (476, 477). Nonetheless, the impact of PSC on the immune system is still debated, as reverting activated to resting PSC appears superior to PSC elimination (478–480) (Figure 9C).

Taken together aPSC/CAF account for the dense stroma formation and ECM modulation. The DR provides a barrier for immune cells, but also for chemotherapy by poor drug access (481). Beyond this “passive action,” aPSC/CAF contribute to the acquisition of major hallmarks of PaCa via cytokines, chemokines, growth factors, and their receptors that promote tumor cell proliferation and chemoresistance, accelerate intrapancreatic nerve invasion and distant metastatic growth and assist establishing an inflammatory milieu that forces immune destruction (482). aPSC/CAF supply essential nutrients and promote metabolic reprogramming backing tumor cell survival and proliferation (483), which is assisted by aPSC/CAF miRNA (484). These activities are briefly elaborated in the following sections. Despite overwhelming evidences for aPSC/CAF supporting PaCa growth and progression, under selected circumstances they may provide a host defense against the tumor, the hypothesis building on poorer prognosis after HH depletion and in αSMA-ko mice (485, 486).

## Activated Pancreatic Stellate Cells and the Crosstalk with Tumor Cells

**Figure 10:**
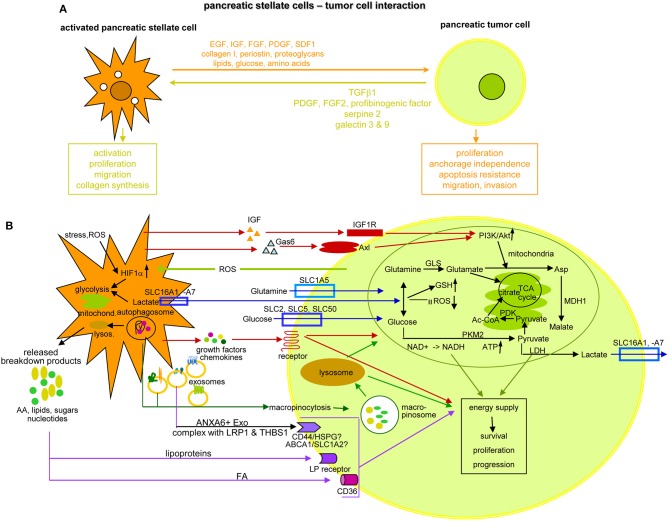
The crosstalk between PSC and pancreatic cancer cells. **(A)** Overview of the support provided by aPSC to PaCa survival, expansion and gain in aggressiveness and feedback by the tumor cell, which sustains PSC activation, expansion and matrix protein synthesis. **(B)** Some of the important components delivered by aPSC toward tumor cells and the initiated changes with a focus on altered metabolism. Glutamate derived from influxed glutamine can replace the TCA cycle to generate citrate, which also can derive from the pyruvate-PDK-Ac-CoA pathway. Lactate, delivered via lactate transporters supports glutamine and glucose generation, GSH upregulation and ROS reduction. Glucose also becomes enriched by glucose transporter in the tumor cell, where PKM2 via NADH and ATP promotes pyruvate generation. After lysosome degradation of aPSC autophagosomes, a plethora of AA, lipids, lipoproteins, sugars, and nucleotides is delivered that in part are taken up by specific receptors, not all being identified so far. Alternatively, autophagosomes are taken up by macropinocytosis, the macropinosome content being delivered after lysosome degradation. Lysosome degradation is also required for the delivery of the aPSC Exo content. Another option is receptor-mediated uptake of selective transmembrane complexes as ANXA6^1^ bound LRP1^1^ and THBS1^1^. The predominant route of transfer from aPSC in PaCa cells is indicated by a color code: red: signaling receptor mediated uptake; blue: delivery or uptake by transporters; vesicle uptake: green; violet: receptor-mediated lipid and lipoprotein uptake; an olive circle encloses for a few of the aPSC-delivered components the pathway, whereby they contribute to the altered metabolism of PaCa cells; others may directly support PaCa survival and aggressiveness. Full name of proteins are listed in Table S1. aPSC support PaCa survival, expansion and progression, which to a considerable degree relies on their input of components initiating energy generation by altered metabolic pathways. Despite the focus on PSC-promoted metabolic adaptation of PaCa cells, the presented data cover only a minor part of the present state of knowledge and additional information can be expected by improved proteomic methodologies combined with organoid cultures.

The extensive crosstalk between aPSC and the embedded tumor cells is pivotal for PaCa survival and progression. Provision of TGFβ, PDGF, FGF2, profibrinogenic factors, serpin2, galectins3, and 9 sustain persisting PSC activation, proliferation, migration, and collagen synthesis. The aPSC also provide growth factors and nutritients (Figure 10A). aPSC/CAF secrete SPARC^1^, involved in cell migration and proliferation (487), and periostin, which modulates invasion via AKT signaling and EMT (488, 489). Most abundant chemokines are CXC/CC family members CCL2/MCP1^1^, CXCL8/IL8, CXCL1^1^, and CXCL2/MIP2^1^, all engaged in PaCa progression (490–492). Increased radioresistance by aPSC/CAF relies on ß1 integrin-FAK activation and DNA damage response regulation (493, 494). An impact on chemotherapy resistance hinges on accessibility (495), activation of the SDF1-CXCR4 axis with subsequent upregulation of IL6, increased HH expression, and IL1β-IRAK4^1^ or mTOR/EIF4E^1^ pathway activation (496–501). Finally, aPSC/CAF support metastasis formation via the HGF/cMET/survivin pathway, which is regulated by TP53^1^/CDKN1A^1^ (502) or through altered lipid metabolism, particularly oleic-, palmitoleic-, and linoleic-acid upregulation (503). Tumor progression is further supported by CAF through partial EMT induction by HH signaling (504) and through aPSC-Exo delivering tumor growth promoting miRNA and lncRNA, which liberate oncogenic/metastasis-promoting mRNA from suppressive miRNA to name only one of the lncRNA functional activities (133). Furthermore, aPSC accompanying migrating tumor cells provide in loco support in establishing premetastatic niches (505, 506).

Nutrient provision by altered metabolic pathways is another important aPSC contribution to PaCa cell progression. This proceeds through increased glycolysis, amino acid (AA) production from protein degradation, by glycosylation and fatty acid synthesis, called the metabolic switch (507). Accordingly, glycolytic enzymes such as hexokinase-2, enolase-2, LDHA, and B^1^ (508) and glycolytic metabolites are elevated (509). In addition, mitochondria adapt and account for energy supply. We recommend a most informative report on the different options, which tumor cells use to alter metabolic pathways (510), and give some examples on specific aPSC contributions. First, aPSC deliver cytokines that by binding to receptors initiate signaling cascades toward activation of the PI3K/Akt pathway, which is central for glycolysis, ATP level maintenance, stabilization of the mitochondrial potential, and tumor cell survival. Two examples are aPSC-derived IGF binding to the IGF1R and Gas6^1^ binding to Axl. Gas6 is a member of the vitamin K-dependent protein family that resembles blood coagulation factors rather than typical growth factors (511). Both, IGF and Gas6 binding promote via PI3K/Akt activation Asp provision (512). Second, uptake of glucose and essential AA is facilitated by transporters either for delivery by aPSC or for uptake by PaCa cells that may also expulse unwanted byproducts, transporter families and their activities being profoundly reviewed (513). An example are glutamine transporters, which are supported by the glutamine-utilizing enzymes glutaminase GLS1^1^, phosphoribosyl pyrophosphate synthetase PRPS2^1^, and carbamoyl-phosphate synthetase 2 CAD converting glutamine to glutamate. Glutamate cannot exit and its accumulation replaces the TCA (tricarboxylic acid) cycle to generate citrate, which also can derive from the pyruvate-PDK-Ac-CoA pathway. Glutamate also stimulates cysteine uptake. Lactate, delivered via lactate transporters supports glutamine and glucose generation, GSH upregulation and ROS reduction. Glucose transporters in the tumor cells further assist glucose enrichment. Promoted by PKM2, NADH, and ATP support the generation of pyruvate. Excellent reviews unravel the current state of knowledge on the TCA cycle and the mitochondrial contribution in detail (508, 514–517). Autophagy accounts for a third support by CAF for nutrient supply. Autophagy is a cytoplasmic recycling process, where unfolded macromolecules, dysfunctional aggregates and organelles are sequestered in a double membrane organelle, called autophagosome, which fuses with lysosomes (518). The released breakdown products, AA, FA, nucleotides, and sugars are reused or released. One of the released AA, alanine is converted into pyruvate that is integrated into the TCA cycle (519). As far as aPSC deliver autophagosomes rather than the single components generated by lysosome degradation, autophagosomes are taken up by macropinocytosis, the nutrients becoming available after degradation in the tumor cell's lysosomes (520). Lysosome degradation is also required for access to nutrients provided by aPSC-derived Exo that modify the metabolic machinery of cancer cells increasing glycolysis and glutamine-dependent reductive carboxylation by providing AA, lipids, and TCA cycle intermediates (521). Finally, PaCa cells essentially depend on large amounts of lipids. FA uptake proceeds via different pathways. Besides gaining access by lysosome degradation of autophagosomes and Exo, the fatty acid translocase CD36 transports circulating free FA across the cell membrane (522, 523). FA sequestered in lipoproteins can be released by low density lipoprotein receptors before uptake by CD36. Alternatively and more frequently in PaCA, lipoproteins are internalized via LDL receptor-mediated endocytosis or macropinocytosis (524, 525). Notably the Exo transfer requires ANXA6+ Exo derived from CAF, where ANXA6 forms a complex with LRP1^1^ and THBS1^1^, the complex being only recovered in aPSC from patient with PaCa (526) (Figure 10B). Thus, though free nutrients are rare in the stroma, embedded aPSC provide a potent source.

In brief, PaCa cells express surface molecules and secrete factors that sustain PSC activation and expansion. aPSC, in turn, support PaCa proliferation, survival and progression. They promote proliferation and migration via cytokine and chemokine delivery, and apoptosis/drug resistance as well as a shift toward EMT via integrin and RTK activation. Ample provision of nutrients supports tumor cell survival and expansion mostly by sustaining altered metabolic pathways. Exo delivered by aPSC add to nutrient supply. Exo miRNA and lncRNA contribute to inactivation of tumor suppressor and liberation of metastasis-associated gene mRNA. lncRNA additionally support chromosome accessibility and transcription initiation, which adds to access of metabolism driving genes. Obviously, stress signals from PaCa cells suffice for aPCS/CAF responding with a plethora of supports.

## Angiogenesis in Pancreatic Cancer

PaCa cells can support angiogenesis (527–529) and microvessel density after PaCa resection correlates with recurrence and poor survival (530). Nonetheless, PaCa are mostly hypovascular and hypoxic, due to a dominance of negative angiogenesis modulators (531, 532).

Several angiogenesis inhibitory factors, elegantly reviewed by Walia et al. (533), are enriched in PaCa. They originate from ECM degradation, poor vascularization being a secondary phenomenon to the fibrotic microenvironment (534). Angiostatin, a 38-kDa tumor cell-derived plasminogen fragment, inhibits primary and metastatic tumor growth by blocking angiogenesis (535–537). Fibstatin, another endogenous angiogenesis inhibitor, is a FN fragment containing the type III domains 12–14 (538). Fibstatin cooperates with CXCL4L1/PF4V1^1^, inhibiting EC proliferation, migration and tubulogenesis *in vitro* and both angiogenesis and lymphangiogenesis *in vivo* (539). Endostatin, another matricellular protein regulating cell function without contributing to ECM structural integrity (533), is a collagen XVIII fragment (540, 541). MMP12 is engaged in endostatin and angiostatin generation (542), VEGF and FGF2 support secretion (543). Endostatin binds both endogenous angiogenesis inhibitors thrombospondin-1 and SPARC (544, 545) and upregulates thrombospondin-1 expression (546). Endostatin also binds VEGFR2 on EC and VEGFR3 on lymphatic vessels preventing activation and downstream signaling (533, 547, 548). By occupying integrin-ECM binding sites, initiation of the tyrosine phosphorylation cascade, src activation, and EC migration are interrupted (549, 550). Endostatin additionally prevents clustering with caveolin-1 and downstream signaling activation (551). A different mechanism underlies the antiangiogenic effect of RNASET2^1^. Independent of its ribonuclease activity, RNASET2 arrests tube formation, accompanied by disruption of the actin network. The authors suggest RNASET2 competing or cooperating with angiogenin (552). Statins, HMGCR^1^ inhibitors, interfere with angiogenesis via VEGF downregulation. Moreover, statins prevent adhesion to the ECM by blocking intercellular adhesion molecules (553). There is, at least, one exception to angiogenesis/lymphangiogenesis inhibition by the PaCa stroma. Stroma embedded mast cells enhance angiogenesis by inducing pro-angiogenic VEGF, FGF2, PDGF, and angiopoietin-1 expression (554).

It may appear surprising that angiogenesis inhibition is a special features of most malignant PaCa with an intensive desmoplasia leading to hypoxia and nutrition deprivation. However, there is no evidence of cell death. PaCa being most well-equipped to cope with nutrient deficits, already outlined in the preceding section, only PaCa cell autonomous programs will be added here. Reuse of vesicle-enclosed nutrients can be liberated in the PaCa cell lysosomes (520). PaCa cell also make use of autonomous autophagy driven by a transcriptional program. Master regulators in converging autophagic and lysosomal functions are MITF^1^ and TFE^1^. A prerequisite for fulfilling these distinct functions relates to their shuttling between the surface of lysosomes, the cytoplasm, and the nucleus in response to nutrient fluctuations and various forms of cellular stress. Shuttling depends on changes in the phosphorylation of multiple conserved amino acids, phosphorylation being mainly promoted by mTOR, ERK, GSK3, and AKT, and dephosphorylation by calcineurin (555, 556). Furthermore, in contrast to most non-transformed tissue, tumor cells engage in *de novo* FA synthesis under hypoxic conditions (517, 557). This occurs particularly when the PI3K-Akt-mTOR pathway is constitutively active as in PaCa. mTOR signaling activates transcription factors of the sterol-regulatory element-binding protein family, which induce expression of the lipogenic genes ACACA^1^, FASN^1^, and SCD^1^ (558, 559).

Taken together, hypoxia-dependent and -independent mechanisms of metabolic reprogramming account for poor vascularization not hindering PaCa progression. Metabolic reprogramming is predominantly promoted by aPSC/CAF and their Exo and is supported by tumor cell autonomous programs.

## Neural Invasion in Pancreatic Cancer

**Figure 11:**
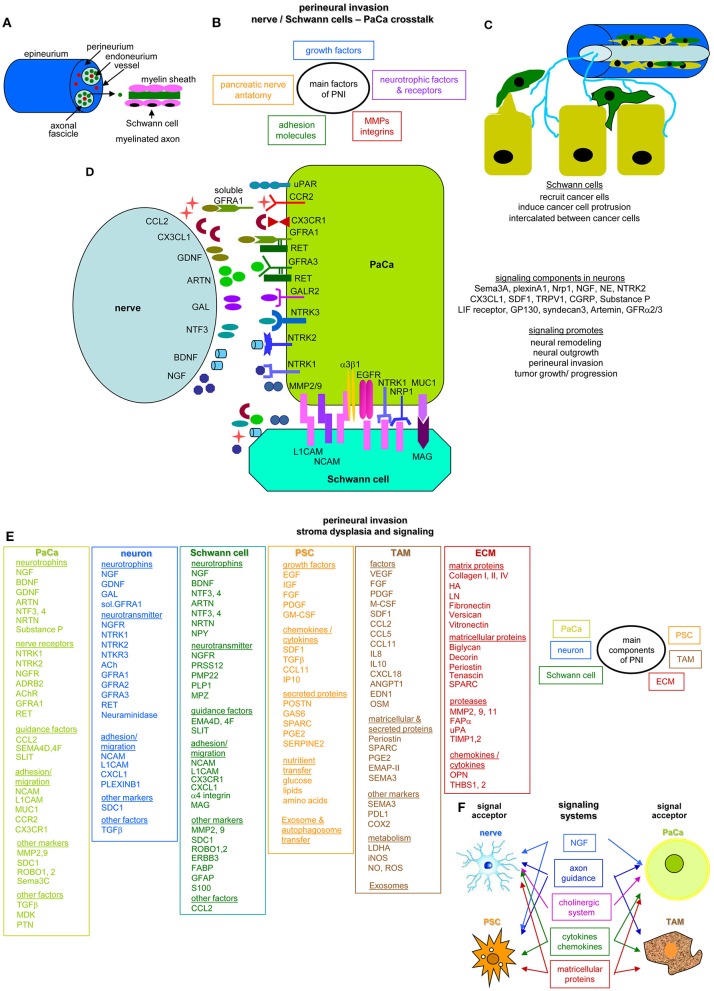
The nervous system and perineural invasion in pancreatic cancer. **(A)** Overview of nerve anatomy. The endoneurium surrounds all axons and serves to separate individual nerve fibers. The axons are covered by Schwann cells, where Schwann cells myelinate the axons. Non-myelinating axons mostly ensheath multiple small caliber axons. **(B)** The anatomy of the pancreatic nerves, neurotrophic factors and receptors as well as growth factors expressed by the engaged cells all contribute to perineural invasion and are supported by adhesion molecules and proteases as demonstrated in **(C)** for Schwann cells that intercalate between tumor cells promoting destruction of the adhesive matrix and actively recruiting tumor cells toward the nerve by signaling via adhesion molecules that promote cytoskeleton reorganization associated with acquisition of a motile phenotype. **(D)** Overview of abundantly delivered neurotrophic factors, cytokines, and chemokines by neurons and the corresponding receptors on PaCa tumor cells that promote tumor cell growth and invasion; dominating in the interaction between Schwann cells and tumor cells are L1CAM and NCAM. Besides homophilic binding, they bind integrins and RTK. MAG binding MUC1 on tumor cells mainly contributes to adhesion. For detailed information on signaling cascade initiation in PaCa, please see reviews mentioned in the text file. **(E)** Besides the direct engagement of neurons, Schwann cells and tumor cells, PSC, TAM, and the dysplastic tumor matrix contribute to PNI. Molecules predominantly contributing to PNI are listed. Selective contributions of aPSC rely predominantly on the transfer of nutrients, Exo and autophagosomes; TAM contribute by the delivery of matricellular proteins like EMAP-II and metabolism regulators such as LDHA and iNOS, the ECM supports PNI by embedded matricellular proteins and proteases. **(F)** All engaged cell populations are also acceptors of signaling cascade activators such as NGF, axon guidance cytokines/chemokines, and matricellular proteins. Activation of the cholinergic system is of major relevance for nerves and tumor cells. Full name of proteins are listed in Table S1. PNI is one of the dominating pathways of PaCa invasion. It is supported by neurotrophins and neurotransmitters delivered by neurons and Schwann cells, the latter in addition providing guidance factors and membrane integrated proteins that promote adhesion and migration. aPSC are essential in nutrient transfer and TAM provide cue enzymes to cope with ROS and NO. TAM and the ECM contribute by matricellular proteins and proteases that facilitate PaCa cell migration toward the nerve.

Innervation of the digestive tract is composed of the intrinsic, enteric nervous system, and afferent extrinsic nerves, transferring information to the central nervous system (CNS) and efferent nerves conveying commands from the CNS to the digestive organs (560). The healthy pancreas has an abundant nerve supply. Ganglia (aggregates of neural cell bodies), the intrinsic component of the pancreatic innervation, are randomly distributed throughout the parenchyma. The afferent system, thin unmyelinated fibers run with the parasympathetic vagus or the sympathetic input splanchnic nerves, the cell bodies are located in the spinal or vagal afferent ganglia. Extrinsic parasympathetic fibers derive from the vagus or the stem brain and end in the synapse of the intrapancreatic ganglia. Postganglionic parasympathetic fibers distribute with sympathetic fibers. Postganglionic sympathetic fibers mostly run with blood vessels (561, 562). Innervation is increased in PaCa (563, 564), nerve fibers forming a dense network that interacts with tumor cells and supports tumor growth and dissemination (565–567). In fact, PaCa metastasize by PNI. Also reported in other cancer, with recovery in 80–100% of patients, PNI is most frequent in PaCa and associated with poor prognosis (37, 568–571). PNI is seen in early stages of PaCa (572, 573) and is independent of lymphatic or vascular metastasis (573, 574) (Figures 11A–C).

PNI is defined as the existence of tumor cells in the epineural, perineural and endoneural spaces of the neuronal sheath (566, 575) and results from mutual message transfer between nerves and tumor cells (566). Though not fully elaborated, many contributing components are known. Nerve growth factor family NGF, BDNF^1^, neurotrophin-3 and−4 (576) bind NTRK1/TRKA^1^ with high- and NGFR/p75NTR^1^ with low affinity (577–580), NTRK1 being highly expressed on nerves and tumor cells (581). Glial cell-derived neurotrophic factors GDNF^1^, NRTN, artemin and persephin are secreted by neural tissue and bind to GFRA1-A4 (582). GDNF expression strongly affects PNI in PaCa (583). This relies on RET receptor-mediated activation of downstream RAS, MAPK/ERK, JNK^1^, PI3K/Akt, and NFκB^1^ pathway activation (584–586). Anti-NGF treatment decreased expression of PNI-involved NTRK1, NGFR, TAC1^1^, and calbindin in neural cells, reduced PNI and inhibited metastases in mice (587). The CXCR4-SDF1 axis also contributes to PNI. CXCR4 promotes tumor cell migration toward nerve cells (588, 589) and SDF1 increases NGF expression (588). Shown in an autochthonous model, PNI plays a significant role in initiation and progression of early PaCa stages, inflammation and neuronal damage in the peripheral and central nervous system already occurring in pancreatic intraepithelial neoplasia (PanIN)2, where acinar-derived cells frequently invade along sensory neurons into the spinal cord and migrate caudally to the lower thoracic and upper lumbar regions. Sensory neuron ablation prevents PNI, astrocyte activation, and neuronal damage, suggesting sensory neurons conveying inflammatory signals from the tumor to the CNS. Neuron ablation also significantly delays PanIN. These data indicate a reciprocal signaling loop between PaCa and the nervous system, including the CNS (590). Axon guidance genes semaphorins and plexins also are frequently altered in PaCa. Semaphorin3C increases PaCa proliferation, invasion, and EMT through ERK1/2 signaling pathway activation (591). Semaphorin3D secretion is regulated by AnnexinA2 phosphorylation. It acts autocrine by binding to the coreceptors plexinD1 and neuropilin-1 (592). Parakrine signaling of Semaphorin3D and plexinD1 between tumor cells and neurons mediates increased innervation, PNI and PaCa metastasis (593). Activation of the peripheral sympathetic nervous system (SNS) also assists PNI. In the healthy pancreas the SNS regulates digestive enzyme and endocrine hormone secretion (594, 595). In PaCa, β-adrenergic receptor activation of the SNS contributes to tumor progression via release of norepinephrine and epinephrine (Figure 11D). In view of the abundance of information coupled with many remaining questions we recommend readers particularly interested in PNI some recent, excellent reviews (38, 596, 597).

Beside tumor cells, nerves, Schwann cells, aPSC, TAM, and the ECM contribute to PNI. The contributing components, sorted according to molecular families and subcellular units are listed (Figure 11E). The complex contribution of dysplastic stroma elements to PNI being not fully unraveled, we only mention few examples. Tumor cells, aPSC, and TAM express GPCR β-adrenergic receptors ADRBA1,-A2, -B1, -B2^1^ that signal via the associated trimeric G-proteins (598–600), HIF-1α (601), and ERK/MAPK (574), which in concert promote tumor growth and metastasis (39). aPSC-derived TGFβ induces NGF via the TGFBR1/ALK5^1^ pathway and HGF-cMET activation (602, 603) that contribute to neural plasticity (604). TAM infiltration also correlates with PNI (605), where TAM-secreted IL8 assists PNI through MMP1-PAR1^1^ signaling via ERK1/2 (606). Schwann cells highly express MAG^1^ (607), which is a receptor for abundant mucin-1 on PaCa (608), MAG-mucin-1 signaling promoting PNI (609). Furthermore, PaCa-derived NGF attracts Schwann cells via NGFR/p75NTR (40), which might be interpreted as the recruitment of nerve cells toward the tumor being the first step in PNI (40, 609). Finally, long distance nerve recruitment predominantly depends on Exo/MV (microvesicles) (610, 611) for several cancer (612, 613). This is best explored for glioblastoma-TEX, which are taken up by tumor cells, EC, and Mϕ, but also by healthy neural cells, and microglia (614). Furthermore, non-transformed cell-derived Exo/MV contribute to message transfer. Oligodendrocytes, glial cells in the brain accounting for axon myelination, shuttle messages between myelinating glia and neurons (615, 616) and between neurons (617). Microglia, the brain's Mϕ defense mechanism, also acts via released MV (618). Microglial MV additionally regulate neuronal excitability accompanied by neuronal ceramide and sphingosine production (618). Schwann cells, too, communicate with the peripheral nervous system via Exo (619).

In brief, the review “Splitting out the demons” is concerned about glioblastoma (620), but may well be of general relevance, particularly for PNI in PaCa. The authors demonstrate that the major signaling systems are NGF, axon guidance molecules, cytokines/chemokines, the cholinergic system, and matricellular proteins that are also delivered by several components of PaCa. Searching for signal acceptors in PaCa revealed that tumor cells, nerves, aPSC, and TAM can all be acceptors of these signaling systems creating a malicious feedback loop in PaCa (Figure 11F).

Spurred by the poor prognosis and PaCa-associated pain (620–623) and PNI being an early event in PaCa development, PNI recently received increasing attention (595). For a long time uncovered molecular pathways due to technical difficulties in culturing engaged cellular components and isolating Exo from defined subpopulations may become unraveled in the near future. Success in culturing Schwann cells particularly opens access to a hitherto inaccessible, important contributor. We consider Exo/MV as an additional promising option to interrupt PNI (618), where improved techniques for isolating and characterizing single stroma cell derived Exo will be of great help in deciphering a PNI-forcing contribution. Despite strong progress, supported by elegant autochthonous mouse models, there is still great need to unravel the complex interactions underlying PNI, which is a prerequisite for therapeutic interference (587, 624).

## Pancreatic Cancer and Immunosuppression

Immune cells are abundant in the PaCa stroma (625, 626), but are immunosuppressive (627, 628), whereas effector cells are rare (629). This accounts for the innate and the adaptive immune system.

### NK

NK are discussed as a therapeutic option in PaCa (630, 631). However, several constraints need clarification as NK are reduced in the juxta tumoral area compared to the stroma, possibly due to sequestration by aPSC (632) and NK apoptosis via FASL^1^-positive tumor cells (613). In addition, cytotoxic activity of NK cells is severely impaired (633).

**Figure 12:**
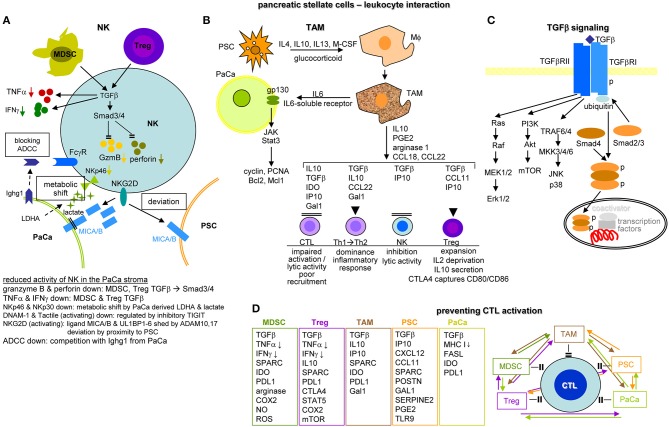
The impact of PSC and tumor cells on immune cells in the pancreatic cancer stroma. **(A)** NK cells in the stroma display reduced activity. This is mainly due to MDSC and Treg that by TGFβ delivery affect TNFα and IFNγ secretion and SMAD3/4 activation, which inhibit GzmB and perforin transcription. The activating NKG2D receptor become deviated toward PSC due to higher expression of MICAB, where MICAB in tumor cells can become shed by ADAM17, free MICAB fragments further deviating NK cells from attacking the tumor cell. The activating receptors NKp46 and NKp30 become downregulated due to a metabolic shift induced by tumor cell derived LDHA and lactate. Activating receptor can also become occupied by inhibitory receptor, like TIGIT. Finally, tumor cells deliver an IgG like molecule, Ighg1, occupying the FcγR of NK cells and thereby interfering with ADCC. **(B)** PSC have a strong impact on driving Mϕ into TAM by the delivery of IL4, IL10, IL13, mCSF, and glucocorticoids. TAM deliver IL6 and soluble IL6 receptor binding to gp130 on tumor cells, which activates the JAK/Stat3 pathway promoting tumor cell survival and expansion by cyclin, PCNA Bcl2, and Mcl1 expression. TAM also affect the activity of additional immune cells. Lytic NK cell activity becomes inhibited by TGFβ and IP10. A shift of Th1 to Th2 is supported by TGFβ, IL10, CCL22, and Gal1. Expansion and activity of Treg is assisted by TGFβ, IP10, and CCL11. Finally, CTL recruitment, activation and lytic activity are impaired by TAM-derived TGFβ, IL10, IP10, IDO, and Gal1. **(C)** A central role of TGFβ in immune deviations relies on binding to the TGFβRII, which promotes RAS, PI3K, and TRAF6/4 pathway activation and on TGFβR1 binding, where phosphorylated Smad4 forms a complex with Smad2/3, the complex migrating into the nucleus promoting together with additional coactivators and transcription factor besides other transcription of NOS, PAI-1 and PDGF. **(D)** CTL activation is prohibited by tumor cells, PSC and immunosuppressive MDSC, Treg and TAM. The major inhibitory factors and membrane molecules are listed. PSC particularly contribute via POSTN, GAL1, SERPINE2, PGE2, and TLR9. Low level MHCI expression on tumor cells hampers CTL activation, high FASL expression contributes to CTL lysis and IDO and PDL1 are inhibitory receptors. As shown in the overview diagram, preventing CTL activation is the result of coordinated activities between all contributing components. Full name of proteins are listed in Table S1. The dense stroma and poor angiogenesis may hamper leukocyte recruitment. However, there is no paucity of immunosuppressive leukocyte, such that changes in metabolism and activation of signaling cascades are dominating immunosuppression. Feedback circles between all contributing elements create a self-replenishing vicious circle.

Activated NK cells bind via activating receptors NKG2D^1^, NKp30^1^, and NKp46^1^ to their ligands major histocompatibility complex class I-related chain MICA/B^1^ and ULBP1-6^1^ (634). NKG2D having a very short cytoplasmic tail uses the adaptor molecules DAP10^1^ and/or DAP12^1^ to initiate downstream signaling (635). In addition, activated NK cells secrete IFNγ, TNFα, GM-CSF^1^, the chemokine ligands CCL1-5^1^, and CXCL8^1^, which trigger activation and recruitment of other innate and adaptive immune cells, broadening and strengthening anti-tumor immune responses (636). In PaCa, instead, decreased NK activity is accompanied by low level NKp46, NKp30, granzymeB, and perforin expression (637). Lactate, a by-product of tumor metabolism also causes NKp46 downregulation (638). Another important group of NK receptors are nectin and nectin-like binding molecule DNAM1^1^. DNAM1 downregulation on NK correlates with PaCa progression (639). Furthermore, though MICA/B is expressed in >70% of PaCa, it is also expressed on PSC (640). NK cells preferentially migrating toward PSC become sequestered in the stroma before reaching the tumor nodules (641). Moreover, ADAM10 and ADAM17 cause shedding of MICA/B and PSC inhibit NK cells via IL6 (642). Finally, NK cells tend to target (Pa)CIC due to enhanced MICA/B expression (643). In view of the CIC plasticity, it remains to be explored, whether CIC targeting by NK is of therapeutic benefit (Figure 12A).

Due to preferentially targeting tumor cells, NK-based immunotherapy was discussed just few years after their discovery (644), hope being fostered by their contribution to antibody-dependent cellular cytotoxicity (645). Further unraveling the impact of their surrounding, efficient use of NK cytotoxic potential may become reality in PaCa.

### Mϕ

TAM are increased in the PaCa stroma (646), high numbers being associated with poor prognosis (647–649). TAM mostly exhibit the suppressive phenotype of CD163+ and CD204+ M2 (650, 651), M2 differentiation being supported by tumor- and Treg-derived IL4, IL10, and IL13 (652). TAM suppress the adaptive immune response via TGFβ, IL10, CCL17, CCL18, CCL22, and PDL1^1^ secretion (653, 654). In addition, CCL2 and CCL20^1^ through chemokine receptor CCR6^1^ binding promote MMP9 upregulation and thereby invasiveness (655, 656) and can contribute to EMT (657, 658). In PaCa, TAM also secrete the serine protease FAP^1^, which stimulates CAF (659) and induces CDA^1^, contributing to drug resistance by metabolizing the active to the inactive form of Gemcitabine (660).

Briefly, the main feature of TAM is the shift to and the preponderance of immunosuppressive M2 in PaCa. Besides suppressing adaptive immune responses, TAM promote CAF and in a positive feedback loop Treg expansion. TAM also strengthen the aggressiveness of PaCa and support drug resistance. Reviews are recommended for a comprehensive overview of special TAM features in PaCa (661, 662) (Figures 12B,C).

### MDSC

MDSC are a heterogeneous group of cells, characterized by myeloid origin, immature state and mostly functional activity. Two subgroups, defined as monocytic (M) and granulocytic (G) MDSC are differentiated by Ly6C^high^ (M-MDSC) or Ly6G^high^ (G-MDSC), M-MDSC exerting stronger suppressive activity (663–665). MDSC are abundant in the PaCa stroma (666). MDSC are recruited toward PaCa via CAF-derived CXCL12 and tumor-derived GM-CSF (588, 667). MDSC hamper T-cell recruitment and activation, which are their major targets and promote Treg expansion (668, 669). MDSC expansion is expedited by M-CSF^1^, GM-CSF, SCF^1^, IL6, IFNγ, IL1β, VEGF, HSP72, IL13, C5a^1^, PGE2^1^, and S100A8/A9 (664, 670). Inhibition of differentiation into mature myeloid cells is spurred by downstream activation of the JAK^1^-STAT3/STAT5 pathway with stimulation of cyclinD1, BCLXL^1^, survivin, c-myc, and S100A8/A9. CCL2 and SDF1 support MDSC recruitment, GM-CSF plays a major role in inflammatory milieu maintenance (667). Prominent signaling molecules engaged in MDSC activity are STAT3, COX2, HIF1α, C/EBPB^1^, HMOX1^1^, and IDO^1^ (654, 670, 671). MDSC interfere at several levels with immune response induction (672). Downstream effector molecules arginase-1 and iNOS^1^ account for L-arginine depletion and ζ-chain downregulation in T-cells (673). iNOS-induced NO and ROS inhibit T-cell proliferation and promote apoptosis. HMOX1 hampers T-cell proliferation by CO production (670, 674). Membrane-bound TGFβ1 assists NK anergy (675). IL10 and TGFβ foster Treg expansion, which become recruited by CXCL10 (676). TGFβ and IL10 also account for IFNγ downregulation (670, 674). IL10 promotes TH2 deviation (677) and M2 polarization (678). Finally, MDSC Exo characterization uncovered MDSC activities being efficiently transferred by Exo (679–681).

Thus, MDSC hamper mostly T-cell, but also B-cell (682) and NK activity, at least in part by supporting Treg expansion and activation. There are several well-established options to combat MDSC induction and activities, frequently used in combination with chemotherapy whose efficacy increases by eliminating MDSC-promoted drug resistance (683, 684).

### Dendritic Cells

Dendritic cells (DC) are professional antigen presenting cells, directly linking the innate and adaptive immune systems, where particularly Th activation essentially depends on processed antigen peptide presentation (685–687) and costimulatory signals provided by DC (688, 689). However, DC activity is severely impaired in cancer (690, 691). In the PaCa stroma, DC are rare and mostly located at the edge of the tumor (692). DC maturation and activation is also hindered by confrontation with immunosuppressive cytokines TGFß, IL6, IL10, and GM-CSF, which activate the STAT3 pathway (693–695). Furthermore, costimulatory molecule CD40 and CD80 expression is reduced in DC, hampering T-cell activation (696). Instead, DC produce CCL22, which recruits Treg (697, 698). Several options for coping with the DC deficit are clinically evaluated, mostly based on the transfer of antigen/peptide-loaded DC, where in PaCa mucin1 and Wilms tumor protein are promising antigen candidates. Loading DC with the patient's TEX is another option that guarantees presentation of the individual tumor's immunogenic antigens/peptides (699–701). The finding that DC-derived Exo are equipped to stimulate T-cells (702), spurred research focusing on DC transfer to overcome poor T-cell responses in PaCa (703–705).

Besides supporting Treg recruitment, DC do not actively contribute to PaCa progression. Unfortunately, their paucity in the tumor stroma, impaired antigen processing and presentation and the insufficient costimulatory molecule supply significantly hamper immune response induction. There is hope for circumventing these drawbacks by DC or DC-Exo transfer, the latter having the advantage of a technically easier implementation in the clinic.

### T-Cells

The adaptive immune system, T-cells and B-cells, is the body's most specialized and efficient defense mechanism. B-cells, secreting antibodies, account for the humoral defense, T-cells for the cellular defense, where CD8+ CTL lyse their targets and CD4+ Th provide soluble factors supporting CTL, B-cells and NK. T-cells are rare in PaCa (706) and PaCa actively inhibit CD4+ T-cell proliferation and migration (707). Furthermore, PaCa tumor cells and the stroma skew Th from cell-mediated responses inducing Th1 toward Th2, which might support tolerance induction (708). The shift toward Th2 is assisted by PaCa-delivered IL10 and TGFβ (709) and by CAF-delivered lymphopoietin (710). Furthermore, lower numbers of T-cells in PaCa (706) may rely on aPSC affecting T-cell migration toward the tumor nodules (631). The Th2 cytokines IL4, IL5, IL6, MIP1α, GM-CSF, MCP1^1^, IL17, IP10, and IL1β are dominant and are associated with poor immune responsiveness and a shorter DFS (disease free survival) (711). Moreover, PaCa inhibit CTL activity. PaCa-derived TGFβ interferes with perforin and granzyme expression (712, 713) and PDL1 on PaCa binds PD1^1^ on CTL, spurring T-cell anergy or death (714). There are subtypes of PaCa that display higher T-cell levels, but the tumor evades the immune response due to amplification of PDL1/2 or upregulation of inhibitory cytokines and the JAK/STAT signaling pathway (715). aPSC also stimulate T-cell apoptosis, decrease IL2 and IFNγ secretion by Th1, but increase IL4 and IL5 secretion by Th2, which is linked to galectin-1 expression on PSC (716, 717).

Though mucin-16 tumor antigen-specific CTL were recovered in few long term survivors, supporting the efficacy of CTL in defending the body's integrity (718), PaCa and aPSC skew toward Th2 and promote T-cell anergy and apoptosis, low level T-cell recovery correlating with a poor prognosis (719) (Figure 12D).

### Treg

Treg are CD4+CD25^high^Foxp3+ cells (720, 721). They contribute to immunosuppression via CD152/CTLA4^1^ (722, 723) and TGFβ and IL10 secretion, which affects Th, CTL, Mϕ, NK, and DC (626, 724–726). In PaCa, Treg are already present at the PanIN stage, expand during tumor progression (727, 728) and are preferentially located surrounding the tumor (729). Treg promote EMT (730) and inhibit Th1 and Th17 effector functions (731). Migration toward the tumor is assisted by tumor chemokines and EC addressins and their ligands on Treg (732). PaCa secrete elevated levels of CCR5 ligands/CCL28, which increases Treg chemotaxis (733). EC in the tumor tissue express high level of mucosal VCAM-1, E-selectin and CD116/CSF2RA^1^, which foster Treg transmigration (734). Increased levels of Treg in the circulation (735) and the tumor stroma (731, 735) correlate with poor prognosis.

There are other unmentioned immune deviations related to PaCa. We recommend overviews focusing on cytokines and chemokines (736–739) and additional immunosuppressive molecules (740), where we only mention a few. RIP1 and 3^1^, highly expressed in PaCa, are key mediators of necroptosis, a caspase-independent cell death. Interestingly, while an *in vitro* blockade of the necrosome was accompanied by increased PaCa aggressiveness, *in vivo* deletion was associated with increased immunogenic myeloid and T-cell infiltrates. The authors suggest that this is due to RIP1/3 signaling through CXCL1 ligation of its receptor CLEC4E/Mincle^1^ that is also expressed on TAM. Thus, TAM lose their immunosuppressive features in the absence of either RIP3 or CLEC4E, which is accompanied by regain of immune defense promoting signaling in T-cells (741). A clinical study showed that an IDO1 inhibitor prevented disease progression. IDO1 catalyzes the degradation of tryptophan to kynurenine (742). Tryptophan is essential for T-cells, but kynurenine supports immunosuppression. Accordingly, IDO1 suppresses effector T-cells and NK and promotes induction, activation and recruitment of Treg and MDSC, the signaling pathways differing between leukocyte subsets (743). An elegant study recently reported on Treg signaling in the tumor environment. Tumor Treg undergo apoptosis and apoptotic Treg exhibit stronger immunosuppressive features than live Treg. Treg apoptosis is due to high oxidative stress susceptibility by weak NRF2^1^ Tf and antioxidant system-associated gene expression. Apoptotic Treg-promoted immunosuppression relies on release and conversion of a large amount of ATP to adenosine by CD39 and CD73, and ADORA2A^1^ pathway activation (744). Galectins are another family of secreted proteins contributing to immune evasion in PaCa (745). Galectins have high affinity for β-galactoside residues, sharing a consensus carbohydrate recognition domain (CRD) responsible for glycan binding, most of their biological functions relying on interactions with glycosylated proteins (746). aPSC account for galectin1 secretion and overexpression in the tumor microenvironment (716). Galectin1 recognizes glycoproteins on T-cells, inhibits transendothelial migration and promotes apoptosis of activated Th1 cells, tilting the immune balance toward a Th2 profile. Galectin1 also impairs NK cell recruitment, induces Treg differentiation, M2 macrophage polarization, and MDSC expansion (747, 748), suggesting galectin1 a key driver in immune evasion in PaCa (748). Galectin9 also is crucial for immune deviation in PaCa. Galectin9 is a ligand for dectin1^1^, highly expressed in PaCa Mϕ. Dectin ligation promotes signaling via syk^1^, PLCγ, and the JNK pathway. The dectin1-galectin9 axis is central in directing the differentiation of TAM to a M2-like phenotype, which suffices for reprogramming CD4+ and CD8+ T-cells (749). Finally, we list some reviews helpful as starting information on PaCa-selective metabolic changes that affect immune responses in PaCa (739, 750–754).

Summarizing at least some aspects of immune modulation by the particular stroma reaction in PaCa, PSC/CAF secrete SDF1 that coats the tumor cells and prevents T-cell infiltration (640, 755). PSC also secrete galectin1 forcing T-cell apoptosis and Th2 deviation (716), but recruiting Treg (485) and supporting mononuclear cell differentiation toward MDSC (756), with suppressive myeloid cells being most abundant in PaCa, TAM accounting for 15–20% and MDSC for 5–10% (716, 757). Tumor-derived GM-CSF and MIP2 account for MDSC (716, 757), CSF1 and BAG3^1^ for TAM (757, 758) recruitment and expansion, GM-CSF being also provided by tumor-associated mesenchymal cells (759). Both MDSC and TAM direct suppression through factors and tumor-cell-specific PDL1 expression (625, 760–762). B-cells are recruited via tumor-derived CXCL13 (763). A shift toward M2 via PI3Kγ-activated BTK^1^ in B-cells and TAM supports PaCa growth and progression (764).

Taken together, PaCa and the dysplastic stroma hamper leukocyte infiltration and skew toward immunosuppressive components. This accounts for the non-adaptive and the adaptive immune system. The strong impact of PaCa and the stroma is reflected by low onco-immunotherapy efficacy, which fosters research on combined therapeutic approaches. With 416 reviews total and 86 in the last 18 months, on immunotherapy in PaCa, we apologize not mentioning this aspect, which goes beyond the scope of our trial giving an overview of the particularly dense crosstalk between PaCa and the stroma. Nonetheless, the body's defense mechanism being highly efficient at maintaining health and coping with a wide range of diseases, there is some hope that after unraveling the complex and intertwined contributions of individual components and signaling pathways, immunotherapy may shortly contribute in defeating PaCa (765).

## Conclusion and Outlook

PaCa has a dismal prognosis and incidence is rapidly increasing. This fostered utmost intense research aiming elaborating the underlying mechanisms, which unequivocally demonstrated the lead role of the PaCa stroma, frequently displaying rebound effects on the tumor cells and between the individual stroma elements. These features seriously aggravate pinpointing single molecular mechanisms such that despite strong progress, we are still tickling the top of a non-melting iceberg. In brief,

1. Unlike most cancer, angiogenesis is reduced in PaCa. Pressure from the dense dysplastic reaction may be partly responsible for inadequate angiogenesis. We assume an active contribution of PaCa-TEX, which interfere with EC migration, expansion and sprouting *in vitro* and *in vivo*. The underlying mechanism remains to be clarified. A comparative analysis of the proteome, coding and noncoding RNA of PaCa-TEX and TEX of a strongly vascularized tumor might be a starting point depicting active contributors to poor PaCa vascularization. Irrespective of the suggested PaCa interference with angiogenesis, the stroma provides copious nutrients and redirects the tumor cells' metabolic pathways such that hypoxia-promoted damages are completely waved.
2. PSC/CAF are central for PaCa stroma dysplasia. The dysplastic stroma strongly adds to immune defense deviation and supports PNI. Progress in suppressing the overshooting stroma reaction may be achieved by a profound analysis of signaling/metabolic pathways linked to aPSC. The discussion still being ongoing, we only mentioned few examples of aPSC/CAF-promoted metabolic reprogramming and possible contributions of aPSC/CAF miRNA and lncRNA (483, 484). Nonetheless and despite overwhelming evidences for PaCa-promoting activities of aPSC/CAF, the dysplastic stroma could serve as a protective barrier for the host against the tumor under selected circumstances. Thus, in the growing list of therapeutic reagents interfering with the metabolism and/or signaling cascades in aPSC (766), the option of reverting PSC to their quiescent state by supporting FA synthesis could be of particular interest (767).
3. PaCa shares with many tumors a paucity of immunogenic tumor-associated antigens and excessive tumor-promoted immunosuppression. These drawbacks for immunotherapy are aggravated in PaCa by the dysplastic stroma. As immunosuppressive cells are enriched in the PaCa stroma, the stroma density may not considerably contribute excluding immune cells. In fact, it is within the stroma that immune cells are killed or deviate toward immunosuppression. Tumor immunotherapy with a strong focus on the transfer of activated DC and T-cells to circumvent low tumor antigen immunogenicity, requires in depth elaboration of in loco deviation to find pathways allowing activation of transferred immune cells within PaCa. This also accounts for the transfer of DC-Exo, where physical barriers are no hindrance, and for antibody-based therapies, where BTK activation by binding to FcRγ+ TAM needs to be bypassed. However, as good progress is already achieved in MDSC elimination, there is hope that remaining hurdles may be solved.
4. PNI, though not unique, is the dominant metastatic route already at early stages of PaCa development. Elaboration of underlying mechanisms is aggravated by an active contribution of the neuronal components. Comparative analyses to brain tumors, particularly glioblastoma, may provide hints for unraveling the crosstalk between tumor cells and nerves including Schwann cells and ganglia. With strong evidence for synaptic information transfer by EV, a focus on the impact of nerve-, microglia-, and Schwann cell-derived Exo/MV on tumor cells could help unraveling the neural system contribution in diverting PaCa cells toward this particular metastatic route.
5. Many studies on PaCIC markers and the feedback on the tumor matrix, EC, the adaptive, and non-adaptive immune system point toward these markers severely affecting host matrix and cells. PaCIC markers are engaged in regulation of transcription, activation of signaling cascades, and metabolic shifts, spurring adhesion, migration, and invasion. Abundantly recovered PaCIC markers on TEX contribute to TEX biogenesis including loading, target binding, and TEX uptake (86). Intensifying studies on cooperation-based peculiarities of PaCIC-TEX markers may uncover a central switch in the PaCIC-stroma interplay, allowing for a unifying concept of PaCIC-TEX-based therapies.
6. We apologize for sparse discussion on signaling pathways in the PaCa-stroma crosstalk. First, signaling pathways are often connected and can be mutually affecting. More importantly, *in vivo* studies only depict the overall changes on tumor cells or stroma, even organoid cultures having some limits in depicting individual components. Nonetheless, organoid cultures provide an excellent method for unraveling the complex and mutual interactions between PaCa cells and their surrounding components (768, 769). It can be expected that continuing advancement in organoid research will markedly increase knowledge of the molecular features of the mutual crosstalk between the distinct components and pave the way for large scale therapeutic screenings that may prove reliable for clinical translation (770).
7. Though providing up-to-date references to the date of submission, for the sake of clarity and length we kindly ask scientists working on special topics gathering additional information. This request particularly applies to ncRNA, where multiple targets for most miRNA hamper coordination and the diverse range of lncRNA functions awaits comprehensive examination (86, 136, 139, 771–773). Furthermore, in view of many eminent reviews, we skipped information on therapeutic translation. Finally, we apologize for not citing numerous outstanding studies.

## Author Contributions

MZ planned the organization of the review and wrote the first draft. WM and ZW helped with data collection and corrected the first draft. All authors approved the final version.

### Conflict of Interest

The authors declare that the research was conducted in the absence of any commercial or financial relationships that could be construed as a potential conflict of interest.

## Footnotes

^1^Alphabetic list of gene/protein full names: Table S1.

## Abbreviations

AA: amino acid
a: activated
ADCC: antibody-dependent cellular cytotoxicity
ASC: adult stem cells
BM: basal membrane
BMC: bone marrow cells
CAF: cancer-associated fibroblasts
CIC: cancer–initiating cells/cancer stem cells
CNS: central nervous system
CoCa: colorectal cancer
CRD: carbohydrate recognition domain
CTL: cytotoxic T lymphocytes
DC: dendritic cells
DFS: disease free survival
DR: desmoplastic reaction
EC: endothelial cells
ECM: extracellular matrix
EE: early endosome
EMT: epithelial mesenchymal transition
ER: endoplasmic reticulum
ERM: ezrin, radixin, moesin
eRNA: enhancer lncRNA
ESC: embryonic stem cells
ESCRT: endosomal sorting complex required for transport
EV: extracellular vesicles
Exo: exosome
FA: fatty acid
FB: fibroblast
FN: fibronectin
GPCR: G protein-coupled receptor
HCC: hepatocellular carcinoma
HNRNP: heterogeneous ribonucleoprotein
ICD: intracellular domain
ILV: intraluminal vesicle
kd: knockdown
ko: knockout
LN: laminin
lnc: long noncoding
LNC: lymph node cells
MDSC: myeloid-derived suppressor cell
MET: mesenchymal-epithelial transition
Mϕ: macrophage
MHC: major histocompatibility complex
miRNA: microRNA
MS: mass spectrometry
MV: microvesicles
MVB: multivesicular body
nc: non-coding
NEAA: non-essential amino acids
NK: natural killer cells
Non-CIC: non-metastasizing tumor cells
PaCa: pancreatic cancer
PanIN: pancreatic intraepithelial neoplasia
PNI: perineural invasion
PSC: pancreatic stellate cells
RISC: RNA induced silencing complex
RBP: RNA binding proteins
RNP: ribonucleoprotein
ROS: reactive oxygen species
RTK: receptor tyrosine kinase
SC: stem cells
SNS: sympathetic nervous system
TAM: tumor-associated macrophages
TCA: tricarboxylic acid
TEM: tetraspanin- and glycolipid-enriched membrane microdomain
TEX: tumor exosomes
Tf: transcription factor
Th: helper T cells
TJ: tight junction
Treg: regulatory T cells.
