# Supplementary material for: Ping-Pong—Tumor and Host in Pancreatic Cancer Progression
Source: Front Oncol. 2019 Dec 16;9:1359. doi: 10.3389/fonc.2019.01359 (PMC6927459; doi:10.3389/fonc.2019.01359)
Supplement: Supplementary file 1 [file Data_Sheet_1.PDF]

Ping-Pong – tumor and host in pancreatic cancer progression

Wei Mu[1,*], Zhe Wang[2], Margot Zöller[2]
[1] School of Public Health, Shanghai Jiao Tong University School of Medicine, Shanghai, China, [2] Department of Oncology, The First Affiliated Hospital of Guangdong, Pharmaceutical University, Guangzhou, China

Supplement

Table S1

Proteins and genes: synonym and full name

**Synonym**	**Full name**
ABC transporter	ATP-binding cassette transporters
ABCA3	ATP-binding cassette subfamily A member 3
ACACA/B	acetyl-CoA carboxylases 1 and 2
Ac-CoA	acyl-CoA
ACH3, 4	acetyl-histone H3, 4
ADAM10/17	ADAM metallopeptidase 10, 17 (TACE)
ADGRB1	adhesion GPCR B1
ADGRE1/F4/80	adhesion G protein coupled receptor E1
ADORA2A	adenosine A2a receptor
ADRBA1,-A2, -B1, -B2	β-adrenergic receptors A1, A2, B1, B2
AGO	argonaute
AKT/PKB	AKT serine/threonine kinase
ALDH	aldehyde dehydrogenase
ALIX/PDCD6IP	programmed cell death 6 interacting protein
alpha SMA	alpha smooth muscle actin
AMP	adenosine monophosphate
ANGPT1, 2ANGP1, 2	angiopoietin 1, 2
ANGPTL4	angiopoietin-like 4
ANXA2, 6	annexin A2, 6
APC	APC regulator of wnt signaling
APP	amyloid precursor protein
ARTN	artemin
AXL	AXL receptor tyrosine kinase
BAD	Bcl2 associated agonist of cell death
BAG3	BCL2 associated athanogene 3
BCL2	BCL2 apoptosis regulator
BCLXL	apoptosis regulator bcl2 like1
BDNF	brain-derived neurotrophic factor
BMP	bone morphogenetic protein
BMPR	BMP receptor
BPAG1/BP230	bullous pemphigoid antigen 1
BPAG2/BP180	180kDa bullous pemphigoid antigen 2/collagen type XVII alpha1 chain
BTK	Bruton’s tyrosine kinase
C/EBPB	CCAAT enhancer binding protein beta
C5	complement component 5
CAD	carbamoyl-phosphate synthetase 2
CALB1	calbindin
CBP/p300	CREB binding protein
CCL1	C-C motif chemokine ligand 1
CCL11	C-C motif chemokine ligand 11
CCL17/TARC	C-C motif chemokine ligand 17/ß chemokine TARC
CCL18/MIP4	C-C motif chemokine ligand 18/ macrophage inflammatory protein 4
CCL2/MCP1	C-C motif chemokine ligand 2/monocyte chemoattractant protein-1
CCL20/MIP3α	C-C motif chemokine ligand 20/macrophage inflammatory protein-3α
CCL22/MDC	C-C motif chemokine ligand 22/macrophage-derived chemokine
CCL3/MIP1A	C-C motif chemokine ligand 3/macrophage inflammatory protein 1α
CCL4/MIP1B	C-C motif chemokine ligand 4/macrophage inflammatory protein 1β
CCL5/RANTES	C-C motif chemokine ligand 5/ß chemokine RANTES
CCNA, B1, E	cyclin A, B1, E
CCR2	C-C motif chemokine receptor 2
CD106/VCAM1	vascular cell adhesion molecule
CD116/CSF2RA	colony stimulating factor 2 receptor alpha subunit
CD11b/ITGAM	αm integrin chain
CD133/PROM1	prominin 1
CD144/CDH5	VE-cadherin
CD146/MUC18	melanoma cell adhesion molecule
CD151	Tspan24
CD152/CTLA4	cytotoxic T-lymphocyte associated protein4
CD169/SIGLEC1	sialic acid binding Ig-like lectin 1
CD171/L1CAM	L1 cell adhesion molecule
CD178/FASL	Fas ligand
CD184/CXCR4	C-X-C motif chemokine receptor4
CD326/EPCAM	epithelial cell adhesion molecule
CD36/PAS-4	platelet glycoprotein 4/fatty acid translocase
CD39/ENTPD1	ectinucleoside triphosphate diphosphohydrolase 1
CD44s	CD44 standard isoform
CD44v6	CD44 variant isoform 6
CD54/ICAM1	intercellular adhesion molecule 1
CD56/NCAM	neural cell adhesion molecule
CD62E/SELE	E-selectin
CD73/NT5E	5’-nuleotidase ecto
CD87/UPAR	urokinase receptor
CD9	Tspan29
CDA	cytidine deaminase

Table S1 continued

Synonym	Full name
CDC37, 42	cell division cycle 37, 42
CDK1	cyclin dependent kinase 1
CDKN1A/P21	cyclin dependent kinase inhibitor 1A
CGRP/CALCA	calcitonin related polypeptide alpha
CIAP1, 2	baculovirus IAP repeat containing 1, 3
CLDN	claudin
CLEC4E/MINCLE	C-type lectin domain family 4 member E
CLN3	lysosomal/endosomal transmembrane protein battenin
cMET	MET proto-oncogene
COX2	cytochrome c oxidase subunit II
CREB1	cAMP responsive element binding protein 1
CRK	crk proto-oncogene, adaptor protein
CSF1/MCSF	colony stimulating factor 1/ macrophage colony-stimulating factor
CSF2/GMCSF	colony stimulating factor 2/granulocyte-macrophage colony-stimulating factor
CSNK1A1/CK1	casein kinase 1 alpha 1
CTNNB1	β-catenin
CX3CL1/NTN	C-X3-C motif chemokine ligand 1/neurotactin
CX3CR1	C-X3-C motif chemokine receptor 1
CXCL	C-X-C motif chemokine ligand
CXCL1/GRO1	C-X-C motif chemokine ligand 1/ melanoma growth-stimulating activity
CXCL10/IP10	C-X-C motif chemokine ligand 10/10 kDa interferon gamma-induced protein
CXCL12/SDF1	stroma-derived factor 1
CXCL2/GRO2/MIP2	C-X-C motif chemokine ligand 2/growth regulated protein beta/macrophage inflammatory protein 2α
CXCL4L1/PF4V1	platelet factor 4 variant 1
CXCL5	C-X-C motif chemokine ligand 5
CXCL8/IL8	C-X-C motif chemokine ligand 8
DAG	diaglycerol
DAP10	hematopoietic cell signal transducer
DAP12	Tyro protein tyrosine kinase binding protein
DECTIN1	C-type lectin domain containing 7A
DGCR8	DiGeorge syndrome critical region 8
DICER	dicer 1, ribonuclease III
DNAM1	nectin-like binding molecules DNAX accessory molecule 1
DOA4	ubiquitin-specific protease
DR6	death receptor 6
DROSHA	Drosha ribonuclease III
DVL	dishevelled segment polarity protein
EDN1	endothelin 1
EGF	epidermal growth factor
EGFR	EGF receptor
EIF4E	eukaryotic translation initiation factor 4E
ENO2	enolase 2
EPI	epinephrine
ERBB2, 3/ 4	erb-b2/-b3/-b4 receptor tyrosine kinase
EREG	epiregulin
ETS1	ETS proto-oncogene 1, transcription factor
FABP(3)	fatty acid binding protein (3)
FAK	focal adhesion kinase
FAP	fibroblast activation protein alpha
FASN	fatty acid synthase
FGF2	fibroblast growth factor 2/basic FGF
FGFR	FGF receptor
FN	fibronectin
FOXP3	forkhead box P3
FZD	frizzled class receptor
GAB1	GRB2 associated binding protein 1
GABARAP	GABA type A receptor-associated protein
GAG	glycosaminoglycan
GAL	galectin
GALR2	galanin receptor 2
GAPDH	glyceraldehyde-3-phosphate dehydrogenase
GAS5, 6	growth arrest-specific 5, 6
GATA2	GATA binding protein 2
GDI1	GDP dissociation inhibitor 1
GDNF	glial cell derived neurotrophic factor
GFAP	glial fibrillary acidic protein
GFRA1, 2, 3, 4	GDNF family receptor alpha 1, 2, 3, 4
GLS	glutaminase
GM3	GM3 ganglioside
GP130	IL6 signal transducer
GRB2	growth factor receptor bound protein 2
GRK2	GPCR kinases 2
GRM6	glutamate metabotropic receptor 6
GSH	pyrimidodiazepine synthase
GSK3B	glycogen synthase kinase 3 beta

Table S1 continued

Synonym	Full name
H3K4, 9, 27ME3	H3 lysine 4, 9, 27 trimethylation
HA	hyaluronan
HAS	hyaluronan synthase
HAVCR1/TIM1	hepatitis A virus cellular receptor 1
HAVCR2/TIM3	hepatitis A virus cellular receptor 2
HDAC6	histone deacetylase 6
HER2	erb-b2 receptor tyrosine kinase 2
HES	hes family bHLH transcription factor
HGF	hepatocyte growth factor
HMGCR	3-hydroxy-3-methylglutaryl coenzyme A reductase
HH	hedgehog
HIF1A	hypoxia inducible factor 1 subunit alpha
HK2	hexokinase 2
HMGB1	high mobility group box 1
HMOX1	heme oxygenase 1
hnRNP U /RNAPol II	heterogeneous nuclear ribonucleoprotein U/RNA polymerase II
hnRNPA2B1	heterogeneous ribonucleoprotein A2B1
HSP70, 72, A8/HSC70	heat shock protein 70, 72, A8
HSPG	heparan sulfate proteoglycan
HYAL	hyaluronidase
IDO	indoleamine 2,3-dioxygenase 1
IFNγ	interferon γ
IGF1, 2	insulin like growth factor 1, 2
IGF1R	IGF 1 receptor
IGFBP	IGF binding protein
IHH	indian hedgehog
IL1β, 6, 8, 13	interleukin 1 beta, 6, 8, 13
ILK	integrin linked kinase
iNOS	nitric oxide synthase 2
IRAK4	interleukin 1 receptor associated kinase 4
IRS1, 2	insulin receptor substrate 1, 2
JAG1	Jagged 1
JAK	Janus kinase
JAM	junctional adhesion molecules
JNK	mitogen-activated protein kinase 8
JUN/AP1	Jun proto-oncogene, AP-1 transcription factor subunit
KRAS	KRAS proto-oncogene, GTPase
KREMEN1	kringle containing transmembrane protein 1
LAMC1	laminin subunit gamma 1
LAMP1	lysosomal associated membrane protein 1
LDHA, B	lactate dehydrogenase A, B
LDL	low density lipoprotein
LEF	lymphoid enhancer binding factor
LGR5/GPR49	leucin-rich repeat containing G-protein coupled receptor 5
LIF	LIF interleukin 6 family cytokine
LIFR	LIF revceptor subunit alpha
LinC00152/CYTOR	cytoskeleton regulator RNA
LKB1	large kinase B1
LN332	laminin 5
LPA	lipoprotein a
LPAR1	lysophosphatidic acid receptor 1
LPL	lipoprotein lipase
LPR1, 5, 6	LDL receptor related protein 1, 5, 6
MAG	myelin-associated glycoprotein
MAP2K3, 4, 6/MKK3, 4, 6	mitogen-activated kinase 3, 4, 6
MAPK1/ERK	mitogen-activated protein kinase 1
MAPK14/p38	mitogen-activated protein kinase 14
MDH1	malate dehydrogenase 1
MDK	midkine
MDR	multidrug resistance gene
MFGE8	milk fat globe-EGF factor 8
MICA/B	MHC class I polypeptide-related sequence A, B
MIF	macrophage migration inhibitory factor
MIP1α	macrophage inflammatory protein 1α
MITF	microphthalmia-associated transcription factor
MLCK	myosin light chain kinase
MMP	matrix metalloproteinase
MPZ	myelin protein zero
MST1R/RON	macrophage stimulating 1 receptor
mTOR	mechanistic target of rapamycin kinase
MUC1	mucin 1, cell surface associated
MYC	myc proto-oncogene
NADPH	nicotinamide adenine dinucleotide phosphate
NANOG	Nanog homeobox
NE	norepinephrine

Table S1 continued

Synonym	Full name
NEU1	neuraminidases 1
NFAT	nuclear factor of activated T cells
NFκB	nuclear factor kappa B
NGF	neural growth factor
NGFR/p75NTR	NGF receptor
NKG2D/KLRK1	killer cell lectin like receptor K1)
NKp30	natural cytotoxicity triggering receptor 3
NKp46	natural cytotoxicity triggering receptor 1
NOTCH1	neurogenic locus notch receptor 1
NPY	neuropeptide Y
NRF2	nuclear factor erythroid 2 like 2
NRP1	neuropilin 1
NRTN	neuroturin
NTF3, 4	neurotrophin 3, 4
NTRK1/TRKA	neurotrophic receptor tyrosine kinase 1
NTRK2/TRKB	neurotrophic receptor tyrosine kinase 2
NTRK3/TRKC	neurotrophic receptor tyrosine kinase 3
OCLN	occludin
OCT4	octamer-binding protein 4
OPN/SPP1	osteopontin
OSM	oncostatin
P53/P21	tumor suppressor protein
PAR1, 2	protease-activated receptor 1, 2
PAUF	pancreatic adenocarcinoma upregulated factor
PCNA	proliferating cell nuclear antigen
PDCD1/PD1	programmed cell death 1
PDGF	platelet-derived growth factor
PDGFR	PDGF receptor
PDK	pyruvate dehyrogenase kinase
PDL1	programmed cell death 1 ligand 1
PGE2	prostaglandin E2
PI3K/PIK3CD	phosphatidylinositol-4,5-bisphosphate 3-kinase/catalytic subunit delta
PI4K	phosphatidylinositol 4-kinase alpha
PKA, C	phosphokinase A, C
PKM2	pyruvate kinase M1/2
PLC, D	phospholipase C, D
PLEC	plectin
PLP1	proteolipid protein 1
PLXNA1, D1	plexinA1, D1
PMP22	peripheral myelin protein 22
POSTN	periostin
POU5F1/OCT4	Pou class 5 homeobox 1/octamer-binding protein4
PPARD	peroxisome proliferator activated receptor delta
PPP1R12A/MYPT1	protein phosphatase 1 regulatory subunit 12A
PRKAA	protein kinase AMP-activated catalytic subunit
PRKD1	protein kinase D1
PRPS2	phosphoribosyl pyrophosphate synthetase 2
PRSS12	serine protease 12
PS	phosphatidylserine
PSEN1	presenilin 1
PSPN	persephin
PTEN	phosphatase and tensin homolog
PTK2B/PYK2	protein tyrosine kinase 2 beta
PTPRK	protein tyrosine phosphatase receptor type K
RAB4	ras oncogene family member B4
RAC	Rac family small GTPase
RAF	raf proto-oncogene
RAGE/AGER	advanced glycosylation end-product specific receptor
RAS	ras proto-oncogene
RET	ret proto-oncogene
RHO	ras homolog family member
RHOGEF/ARHGEF28	Rho guanine nucleotide exchange factor
RIP1, 3	receptor interacting protein 1, 3
RNASET2	ribonuclease T2
RNF43	ring finger protein 43
ROBO1,2	roundabout guidance receptor 1
ROCK	Rho associated coiled-coil containing protein kinase
RSPO	R-spondin
RTKN	rhotekin
RUNX1	RUNX family transcription factor 1
S100	S100 calcium binding protein
S1P	sphingosine-1 phosphate
S1PR1	S1P receptor
SCD	stearoyl-Co desaturase
SCF	stem cell factor

Table S1 continued

**Synonym**	**Full name**
SDC	syndecan
SEMA3A, -3C, -3D, -3E	semaphorin3A, C, D, E
SERPINE2	serine protease inhibitor nexin-2
SGPP1	sphingosine-1-phosphatase
SHH	sonic hedgehog
SLC1A5	solute carrier family 1 member 5
SLIT2	Slit guidance ligand 2
SLIT-ROBO/SRGAP1, 2C	Rho GTPase activating protein 1/ 2C
SLUG	snail family transcriptional repressor 2
SMAD2, 3, 4	SMAD family member 2, 3, 4
SMO	smoothened frizzled class receptor
SNAIL	snail family transcriptional repressor
SNARE	soluble *N*-ethylmaleimide-sensitive fusion attachment protein (SNAP) receptor
SNCA	α-synuclein
SOS	SOS Ras/Rac guanine nucleotide exchange factor
SP1	Sp1 transcription factor
SPARC	serine protein acidic and rich in cysteine
SRC	src proto-oncogene, non-receptor tyrosine kinase
STAB1	stabilin1
STAT	signal transducer and activator of transcription
SYK	spleen-associated tyrosine kinase
SYNCRIP	synaptotagmin binding cytoplasmic RNA interacting protein
SYT	synaptogamin
TAC1	tachykinin precursor-1
TCA	tricarboxylic acid,
TCF	T cell factor
TCF4	transcription factor 4
TF/F3	tissue factor/coagulation factor III
TFE	transcription factor binding to IGHM enhancer
TGF	transforming growth factor
TGFBR1/ALK5	TGFβ receptor 1
TGFB	transforming growth factor β
THBS1	thrombospondin-1
TIE2/TEK	TEK receptor tyrosine kinase
TIM4	T cell immunoglobulin and mucin domain containing
TIMP	TIMP metallopeptidase inhibitor
TLR2, 3, 4, 5, 9	toll-like receptor 2, 3, 4, 5, 9
TNC	tenascin
TNF	tumor necrosis factor
TNFR	TNF receptor
TP53	tumor protein p53
TRAF4, 6	TNF receptor associated factor 4, 6
TRBP	RISC-loading RNA binding subunit
TRCP/BTRC	beta-transducin repeat containing E3 ubiquitin protein ligase
TRPV1	transient receptor potential cation channel subfamily V member 1
TSG101	tumor susceptibility 101
ULBP1-6	UL16 binding protein 1-6
ULK1	unc-51 like autophagy activating kinase 1
UPA	plasminogen activator, urokinase
VCAN	versican
VCL	vinculin
VEGF	vascular endothelial growth factor
VEGFR	VEGF receptor
VIM	vimentin
VWF	von Willebrand factor
WNT	wingless-type transcription factors
WT1	Wilms tumor protein
YAP	Yes associated protein
ZNRF3	zinc and ring finger 3
ZO-1	zonula occludens protein / tight junction protein 1
